# Within-patient gene transfer between transiently and chronically infecting bacteria causes extreme antibiotic resistance during lung infections

**DOI:** 10.1038/s41564-026-02414-3

**Published:** 2026-07-23

**Authors:** Sardar Karash, Hannah L. Betts, Matthew C. Radey, Samuel Lee, Sarah J. Morgan, Barbara J. Waddell, Sara Klee, Beth A. Traxler, Michael D. Parkins, Rafael E. Hernandez, Alison F. Feder, Colin Manoil, Pradeep K. Singh

**Affiliations:** 1 Department of Microbiology, University of Washington, Seattle, WA USA; 2 Departments of Microbiology, Immunology and Infectious Diseases, University of Calgary, Calgary, Alberta Canada; 3 Department of Medicine, University of Calgary, Calgary, Alberta Canada; 4 Department of Pediatrics, University of Washington, Seattle, WA USA; 5 Center for Global Infectious Disease Research, Seattle Children’s, Seattle, WA USA; 6 Department of Genome Sciences, University of Washington, Seattle, WA USA; 7 Howard Hughes Medical Institute, Seattle, WA USA; 8 Division of Public Health Sciences, Fred Hutch Cancer Center, Seattle, WA USA; 9 Department of Medicine, University of Washington, Seattle, WA USA

**Keywords:** Antibiotics, Clinical microbiology

## Abstract

Antibiotic resistance arising during infections is generally thought to be due to mutations in pathogen genomes. Here we studied *Pseudomonas aeruginosa* and *Achromobacter* collected from people with cystic fibrosis and non-cystic fibrosis bronchiectasis that suddenly developed 10,000-fold increases in tobramycin resistance after tobramycin treatment was initiated. Genomic analysis showed that resistance did not arise from mutation accumulation or strain displacement. Instead, it occurred because plasmid-borne resistance genes were transferred to the previously sensitive pathogens inside patients’ lungs. In some cases, we identified the bacteria that carried plasmids into patients’ lungs and they were species capable of environmental growth like *Pseudomonas putida*. The most commonly transferred gene was an *aac(3)* aminoglycoside *N*-acetyltransferase (*aac(3)-IIId*), not previously associated with clinical resistance. Further analysis suggested that this gene was mobilized from environmental bacteria by a transposon and incorporated into transmissible plasmids. This work shows that gene transfer between transiently and chronically infecting bacteria can produce sudden and large increases in antibiotic resistance during human infections.

## Abstract (web summary)

Antibiotic resistance gene acquisition by *Pseudomonas aeruginosa* and *Achromobacter* from transiently infecting bacteria drives rapid and extreme resistance to tobramycin during chronic lung infection within patients.

## Main

Antibiotic resistant infections are a major global health problem^1^. In many cases, infections are initiated by antibiotic-sensitive pathogens, and resistance emerges inside the host after treatment is started^2–4^. Infections in people with cystic fibrosis (PwCF) are a prime example^4^. PwCF have impaired lung host defenses and can develop devastating chronic lung infections with *Pseudomonas aeruginosa* (Pa) and other pathogens such as *Achromobacter* sp. (Ac). These pathogens are often antibiotic sensitive when they first appear, and resistance develops over time inside patients’ lungs^4–6^. Resistance can be highly consequential as it is associated with poor outcomes such as lung function decline, need for lung transplantation and premature death^7^.

Resistance that arises in vivo is generally thought to be caused by the accumulation of mutations in pathogen genes that effect antibiotic modification or permeability, stress responses, or in antibiotic targets^8,9^. Because these genes usually have other critical functions, mutations with conservative effects or those that can be compensated for by secondary mutations are favoured to limit fitness costs^10,11^. As a result, resistance due to mutation accumulation often develops in a gradual, step-wise manner wherein mutations with minor effects emerge, fitness effects are compensated for, and more resistance-producing mutations then accumulate^11–13^.

Here we observed multiple chronic infection cases where the pattern of in vivo resistance development seemed inconsistent with mutation accumulation, so we sought to understand the mechanism responsible. The cases involved people with CF and non-CF bronchiectasis from disparate locations in North America who were initially infected by antibiotic-sensitive Pa and Ac strains. Soon after treatment with the antibiotic tobramycin, ~10,000-fold increases in tobramycin resistance suddenly appeared and then persisted for years. Our work shows that extreme resistance can be caused by the in vivo transfer of resistance genes from transiently infecting bacteria to antibiotic-sensitive pathogens already established in human lungs. This mechanism could markedly compromise antibiotic treatment.

## Results

### Extreme resistance suddenly emerges in patients’ lungs

We studied isolates from 17 patients with chronic lung infections that evolved extreme tobramycin (Tob) resistance (eTobR). We defined eTobR as minimum inhibitory concentrations (MICs) ≥ 1,024 µg ml^−1^; resistance is conventionally defined as ≥4 µg ml^−1^ (ref. ^14^). These extreme resistance levels are relevant because high doses of inhaled tobramycin are repeatedly administered as maintenance treatment for chronic Pa lung infections in CF^15^. Multiple longitudinal isolates were available from 11 patients; 10 had CF and Pa infections; 1 had non-CF bronchiectasis and *Achromobacter* sp. (Ac) infection (Supplementary Table 1). Patients ranged in age from 2–67.5 years and were located in Seattle, Calgary, Pittsburgh and New York City.

**Fig. 1:**
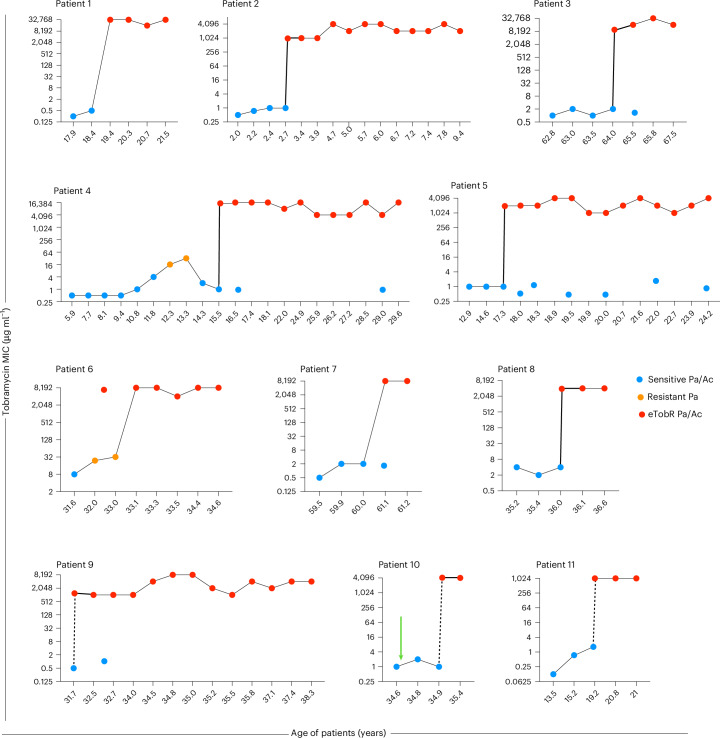
Tob resistance suddenly emerges during established lung infections. Tobramycin (Tob) MICs of longitudinally collected isolates from 11 patients with chronic lung infections as a function of age (timepoints are shown as discrete variables for clarity). Patients 1–7 and 9–11 were people with CF and Pa. Patient 8 had non-CF bronchiectasis and was infected with Ac. Blue points represent Tob-sensitive isolates; orange points, isolates with Tob MICs 16–32 µg ml^−1^; red points, isolates with extreme eTobR (MICs ≥ 1,024). Solid lines indicate MIC trends of clonally related isolates, dashed lines show trends of unrelated isolates; all MIC values are given in Supplementary Table 2. The green arrow indicates the age when patient 10 initiated elexacaftor/tezacaftor/ivacaftor (ETI). Source data

The pattern of resistance development showed several common features. First, the Tob MICs of initial isolates were low; 0.125–8 µg ml^−1^ inhibited growth, and sensitive isolates were observed multiples times before resistance emerged (Fig. 1). Second, extreme resistance suddenly developed after high-dose inhaled Tob was started. Average Tob MICs increased by 9,340-fold (range: 313–65,536-fold) or 8,557 µg ml^−1^ (range 1,024–32,768 µg ml^−1^) over consecutive collection timepoints (Fig. 1). Third, extreme resistance was durable over years, and sensitive isolates were consistently detected after resistance emerged in only one case (Supplementary Table 2).

### Extreme resistance evolved in previously sensitive lineages

**Fig. 2:**
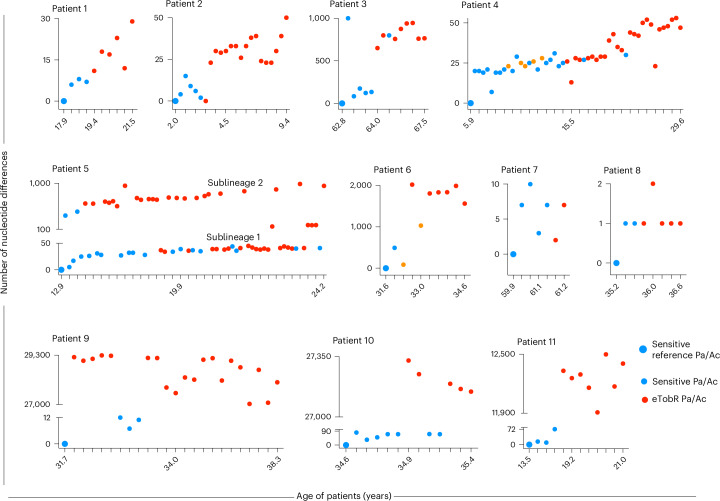
Isolates from patients 1–8 are clonally related. Number of nucleotide differences in all sequenced isolates compared to the earliest isolate from each patient (large blue point). In patients 1–8, all sequenced isolates were clonally related to the initial sensitive isolate. In patients 9–11, eTobR isolates were not clonally related to the sensitive isolates that preceded them (see isolate MLST types in Supplementary Table 1). Patients 3, 6, and sublineage 2 in patient 5 have hypermutator phenotypes (see Extended Data Fig. 1b). Source data

**Extended Data Fig. 1:**
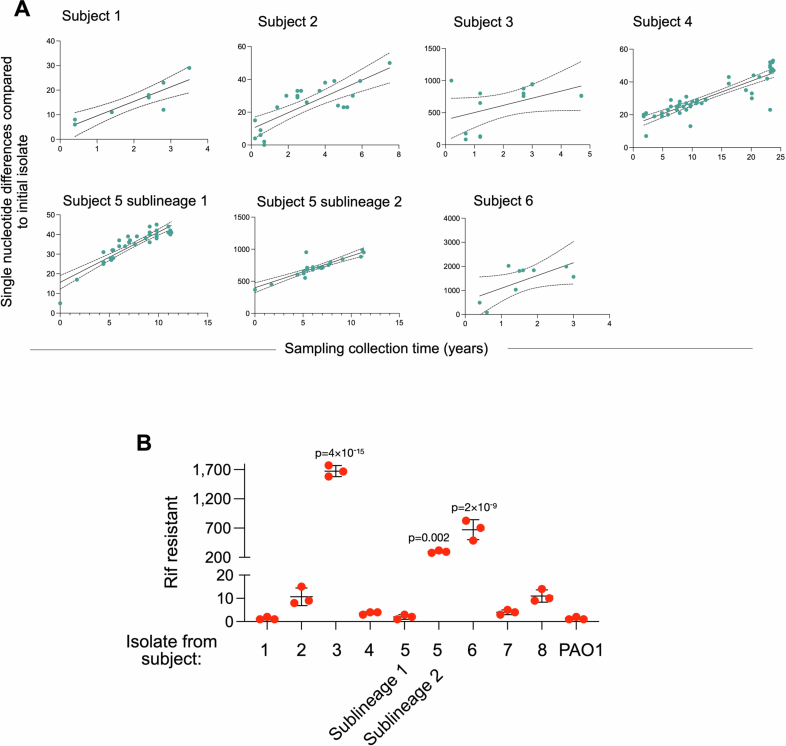
Nucleotide substitution rates and rifampicin hypermutator assay for isolates from subjects 1-8. **(A)** Longitudinally collected isolates from the eight primary subjects were analyzed to estimate nucleotide substitution rates. Subjects 1-7 were infected with *P. aeruginosa* (Pa), and subject 8 with *Achromobacter* sp (Ac). Each point represents a whole-genome sequenced isolate. The first isolate from each subject was used as a reference to identify nucleotide differences in subsequent isolates (see Supplementary Table 2). The plots show the accumulation of nucleotide differences over time; the solid line represents the mean estimate of the fitted linear regression, and dashed lines indicate the 95% confidence intervals. The x-axis indicates years since the first sampling; the y-axis shows the number of accumulated nucleotide differences. Linear regression was used to estimate the rate of mutation accumulation, with the slope representing the number of nucleotide substitutions per year. Statistical parameters of the regression are shown in Supplementary Table 4. Our calculated substitution rates per site per year were similar to those reported for within-host evolution of non-hypermutator *P. aeruginosa* in CF^83^. Substitution rates per site per year were calculated as described in the Methods. **(B)** The first tobramycin-sensitive Pa isolate from subjects 1-7 and Ac from subject 8 were assessed for the hypermutator phenotype. 10^8^ cells from overnight cultures were plated on LB agar containing 300 µg/mL rifampicin. The laboratory Pa strain PAO1 was used as a non-hypermutator control. Data are shown as mean ± s.d. from n = 3 independent replicates per subject. Pa isolates from subject 3, sublineage 2 from subject 5 (see Fig. 2), and subject 6 exhibited a hypermutator phenotype, as they produced significantly higher numbers of rifampicin-resistant mutants compared to the PAO1 control (one-way ANOVA). Source data

We began investigating the resistance mechanism by determining whether resistance developed within pre-existing lineages or whether sensitive strains were displaced. Genome sequencing of banked Pa or Ac isolates from patients 1–8 (minimum coverage ≥10×, mean 81×) showed that each patient’s first-collected Tob-sensitive isolate, and subsequent eTobR isolate had nearly identical gene content (Supplementary Table 3). In addition, except for Pa from patients 3 and 6, and a sublineage from patients 5 that were hypermutators, sequential isolates from most patients differed by only 1–50 nucleotides over the ~6.5 × 10^6^-bp chromosome (Fig. 2 and Supplementary Table 2; see Extended Data Fig. 1a and Supplementary Table 4 for substitution rates). Thus, only a single Pa or Ac strain was identified in each of patients 1–8, so extreme resistance emerged in the previously sensitive pathogen lineage that infected these patients. In contrast, eTobR isolates from patients 9–11 differed by thousands of nucleotides (12,000–28,000) from preceding Tob-sensitive isolates (Fig. 2), indicating strain displacement as the cause in these patients.

We focused mechanistic studies on the clonally related pathogen lineages from patients 1–8 (called ‘primary patients’). We compared the sequences of 51 genes previously implicated in Tob resistance^16–21^ from consecutively collected sensitive and eTobR Pa from each patient and found that all were nearly identical in these isolate pairs (Supplementary Table 5), including in hypermutator lineages (Extended Data Fig. 1b). We considered the possibility that multiple mutations not previously implicated in Tob resistance could act together to produce eTobR. However, consecutive sensitive and eTobR isolate pairs from one patient (patient 8) showed no nucleotide differences over the entire chromosome, which argued against the combined action of multiple mutations (Supplementary Table 6). Thus, we explored other mechanisms.

### eTobR isolates acquired plasmids carrying resistance genes

**Fig. 3:**
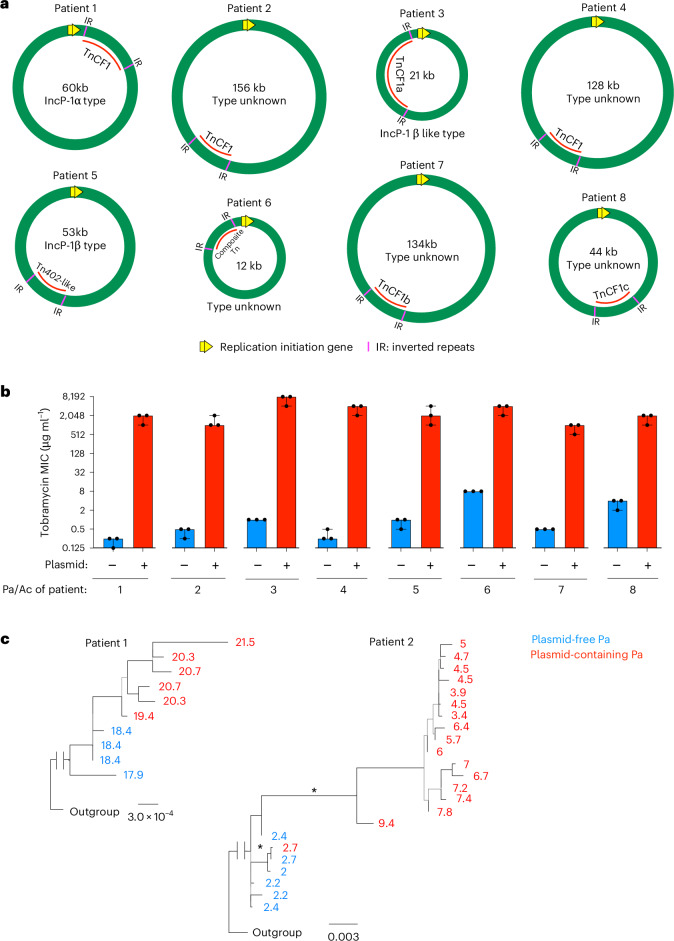
eTobR isolates acquired plasmids. **a**, Diagram of plasmids acquired by the previously sensitive Pa and Ac lineages in patients 1–8. Plasmids in 6 patients carried the TnCF1 transposon or one of its variants (that is, TnCF1 or TnCF1a–c; see Supplementary Table 1 for TnCF1 variants). See NCBI GenBank accessions CP199861, CP199842, CP199831, CP199828, CP199822, CP199817, CP199814 and CP199807 for complete plasmid sequences. The plasmids in patients 2, 4, 6, 7 and 8 were untypeable using plasmid-typing software MOB-suite and PlasAnn (see Methods and Supplementary Table 7). **b**, Plasmid acquisition causes eTobR. The last sensitive Pa from patients 1–7 and last sensitive Ac from patient 8 became Tob resistant after plasmids acquired by the lineage were inserted by conjugation (patients 1, 2, 4, 5, 7 and 8) or electroporation (patients 3 and 6). Data are shown as median ± 95% CI of *n* = 3 independent experiments. Source data. **c**, Phylogenetic trees infer that eTobR Pa were descendants of sensitive Pa. Whole-genome maximum-likelihood phylogenetic trees were constructed from longitudinally collected Pa isolates. The branch tips are labelled with the patient’s age in years at the time of isolate collection; blue tips indicate plasmid-free Tob-sensitive isolates; red tips indicate plasmid-carrying eTobR isolates. Black and grey branches indicate ultrafast bootstrap values ≥90 and <90, respectively. An example of distinct sublineages containing plasmids in patient 2 is marked with ‘*’ and was assessed via parsimony. See Extended Data Figs. 4 and 5 for additional patients. Source data

**Extended Data Fig. 2:**
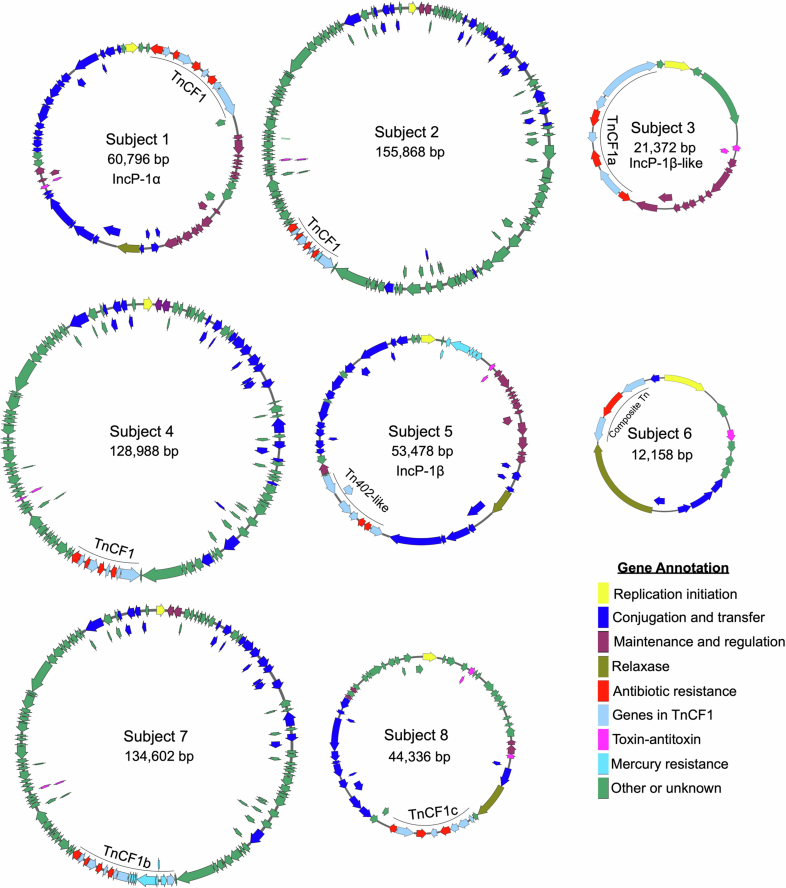
Map of complete plasmids from subjects 1-8. Plasmids from the first eTobR Pa isolates from subjects 1-7 and the first eTobR Ac isolate from subject 8 were sequenced to generate complete circularized plasmids. The first genes on the plasmids are replication initiation genes, shown in yellow. Aminoglycoside resistance is mediated by aminoglycoside-inactivation enzymes located on transposons. Plasmids from subjects 1, 2, 3, 4, 7, and 8 carry TnCF1 transposon or TnCF1 variants (Supplementary Table 1). Plasmid annotation was performed as described in the Methods section.

**Extended Data Fig. 3:**
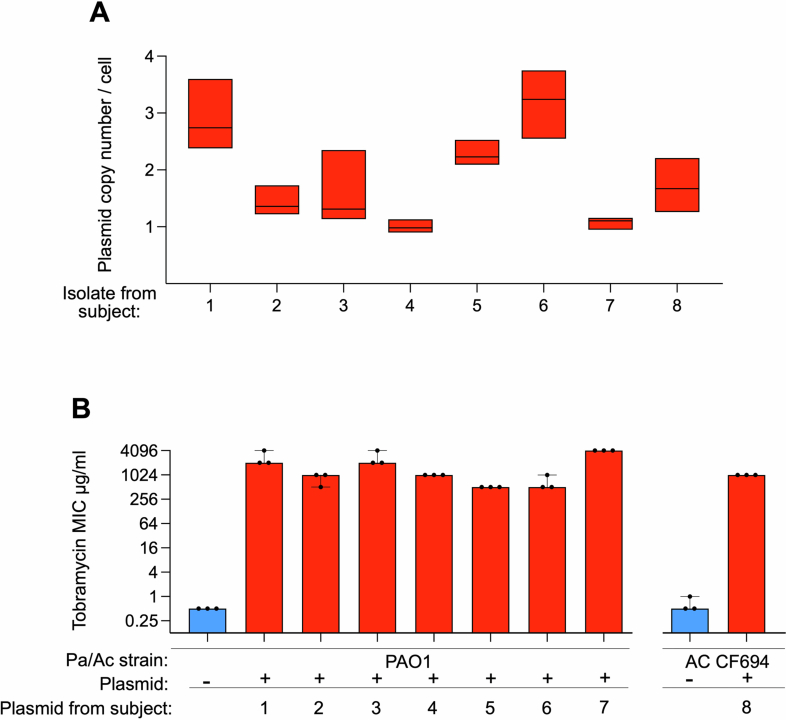
eTobR isolates carry low copy number plasmids and cause eTobR in lab strains. **(A)** Droplet digital PCR (ddPCR) measurements of plasmid copy number in plasmid-containing Pa (subjects 1-7) and Ac (subject 8) isolates. Copy number was measured in 3 clinical isolates collected from each subject at different time points, bars indicate minimum and maximum, lines indicate median. **(B)** Plasmid acquisition causes eTobR. The sensitive PAO1 lab strain and sensitive Ac strain CF694 exhibited marked increases in Tob MICs after plasmids from corresponding subjects (subjects 1-7 PAO1 and subject 8 Ac CF694) were inserted by conjugation or electroporation. Data are shown as median ± 95% CI from n = 3 independent experiments. Source data

We generated completed genomes of patients’ isolates using short and long-read sequencing (Supplementary Table 2) and were surprised to find that eTobR isolates from all primary subjects carried plasmids, whereas the preceding clonally related sensitive isolates did not. Acquired plasmids varied from 12–156 kb (Fig. 3a and Extended Data Fig. 2), had 1–3 copies per cell (Extended Data Fig. 3a), and included conjugative (patients 1, 2, 4, 5, 7 and 8) and non-conjugative (patients 3 and 6) plasmids. Plasmids belonged to IncP1 (subject 1 and 5), IncP1-like (subject 3) and unknown types (patients 2, 4, 6–8) (Supplementary Table 7). Patients 2, 4 and 7 carried the same previously uncharacterized plasmid type (Fig. 3a and Supplementary Table 7). All identified plasmids harboured aminoglycoside-inactivating genes, and in patients 1–4, 7 and 8, resistance was conferred by the previously undescribed *aac(3)-IIId* gene (see below).

We isolated plasmids from the first-detected eTobR isolate from all 8 primary patients and transferred them by conjugation or electroporation into the last-detected Tob-sensitive isolate from that patient (Fig. 3b), and into unrelated Pa (PAO1 for patients 1–7) and Ac strains (Ac CF694 for patient 8) (Extended Data Fig. 3b). Plasmid transfer reproduced the eTobR phenotype in all cases.

### Sensitive isolates acquired plasmids inside patients’ lungs

Our finding that plasmid-free (detected first) and eTobR plasmid-containing isolates (detected later) were clonally related in the 8 primary patients suggests that established Pa and Ac strains acquired plasmids while inside patients’ lungs. However, pathogen gene acquisition inside humans has been infrequently described (see ref. ^2^ and discussion). Thus, we used phylogenetic analysis to investigate the alternative scenario wherein plasmid-containing isolates initiated the infections, but variants that had lost plasmids were detected first (perhaps because plasmid-free variants were more fit).

Phylogenetic analysis could distinguish between these possibilities: The finding that plasmid-containing isolates were genetic descendants of plasmid-free ancestors would indicate that plasmids were acquired within patients’ lungs. The finding that plasmid-free isolates were descendants of plasmid-containing ancestors would indicate that plasmid-containing isolates were acquired first.

**Extended Data Fig. 4:**
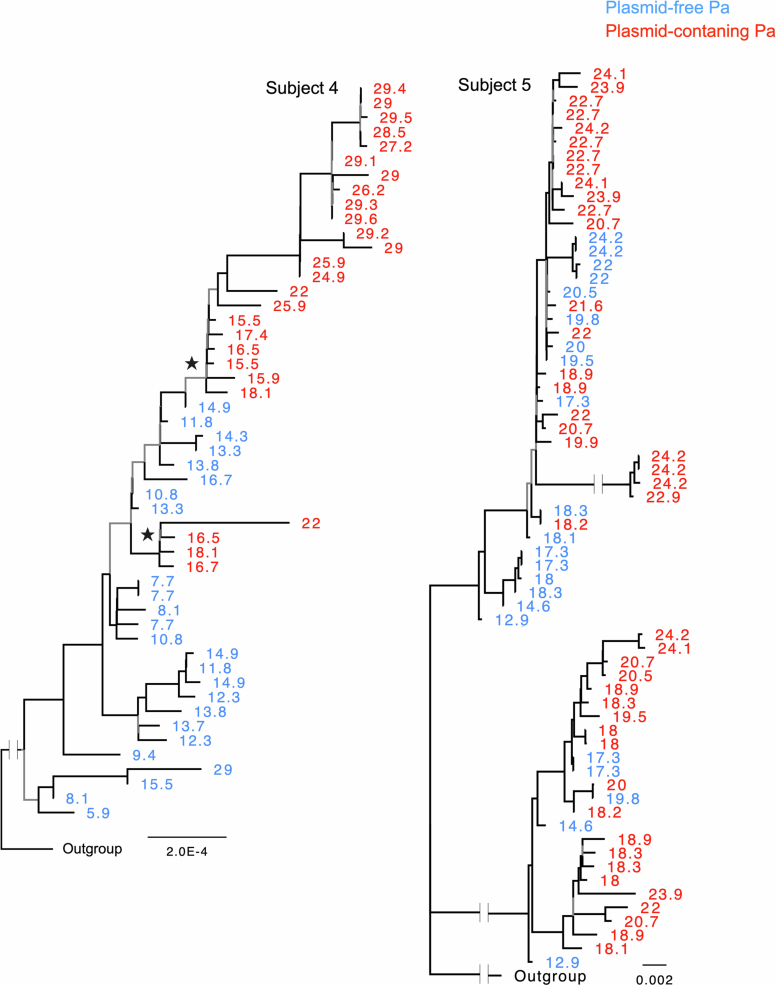
Phylogenetic trees infer that eTobR Pa were descendants of sensitive Pa. Whole-genome maximum likelihood phylogenetic trees were constructed from longitudinally collected Pa isolates. Heterologous Pa isolates ERR953487, and SRR1014306 from NCBI were used for subjects 4 and 5, respectively, for tree rooting. The tips of the branches in these non-temporal trees were labeled with the subject’s age at the time of isolate collection (in years). Blue tips indicate isolates that were Tob-sensitive and did not carry plasmids. Red tips indicate eTobR isolates that harbored resistance plasmids. Branches with black color indicate ultrafast bootstrap values ≥ 90, while gray branches indicate values < 90. Two examples of evolutionarily distinct sublineages containing plasmids in subjects 4 and 2 (see Fig. 3c) are marked with stars (★) and were assessed via parsimony. See Fig. 3c and Extended Data Fig. 5 for additional subjects.

**Extended Data Fig. 5:**
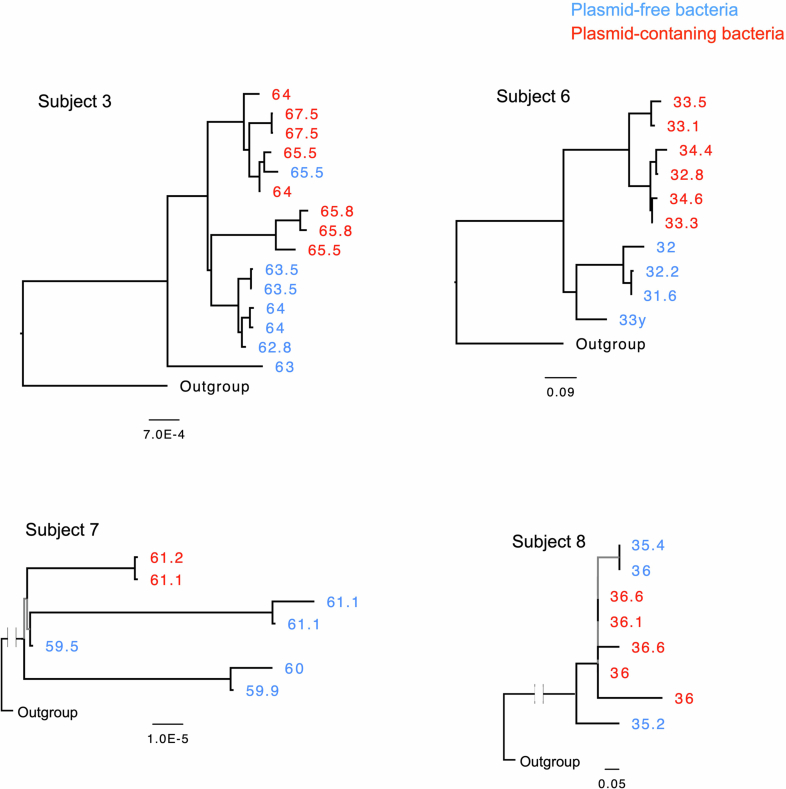
Phylogenetic trees of isolates from subjects 3, 6, 7, and 8, in whom ancestry was not established. Whole-genome maximum likelihood phylogenetic trees were constructed from longitudinally collected Pa isolates. Heterologous Pa isolates SRR1014444, SRR2939340, and SRR14790817 from NCBI were used for subjects 3, 6, and 7 respectively, for tree rooting. Heterologous Ac isolate SRR2821369 from NCBI was used for subject 8 for tree rooting. The tips of the branches in these non-temporal trees were labeled with the subject’s age at the time of isolate collection (in years). Blue tips indicate isolates that were Tob-sensitive and did not carry plasmids. Red branches indicate eTobR isolates that harbored resistance plasmids. Branches with bold black color indicate ultrafast bootstrap values ≥ 90, while gray branches indicate values < 90. See Fig. 3c and Extended Data Fig. 4 for additional subjects.

For all lineages with clear ancestries (patients 1, 2, 4 and 5), maximum-likelihood trees generated independent of isolate collection time and excluding plasmid sequences showed that plasmid-containing isolates were derived from the earlier-collected plasmid-free clones (Fig. 3c and Extended Data Fig. 4). Phylogenetic analysis in other patients could not establish which isolates were ancestral (Extended Data Fig. 5). These data support the conclusion that previously sensitive pathogens acquired plasmids producing eTobR inside infected patients.

This study was not designed to measure plasmid acquisition frequency in patient cohorts. However, we generated a rough estimate from one isolate bank that recorded numerical MICs. Two-hundred and nine children provided Pa to this bank and isolates from 6 children (2.9%) developed eTobR (that is, Tob MIC increased from <8 to ≥1,024 µg ml^−1^ during the period of isolate collection). Resistant isolates from all 6 patients carried eTobR plasmids (see ‘*’ in Supplementary Table 1). Plasmid acquisition could be more frequent in adults with longer infection durations and increased antibiotic use.

### Environmental bacteria carried plasmids to patients’ lungs

**Fig. 4:**
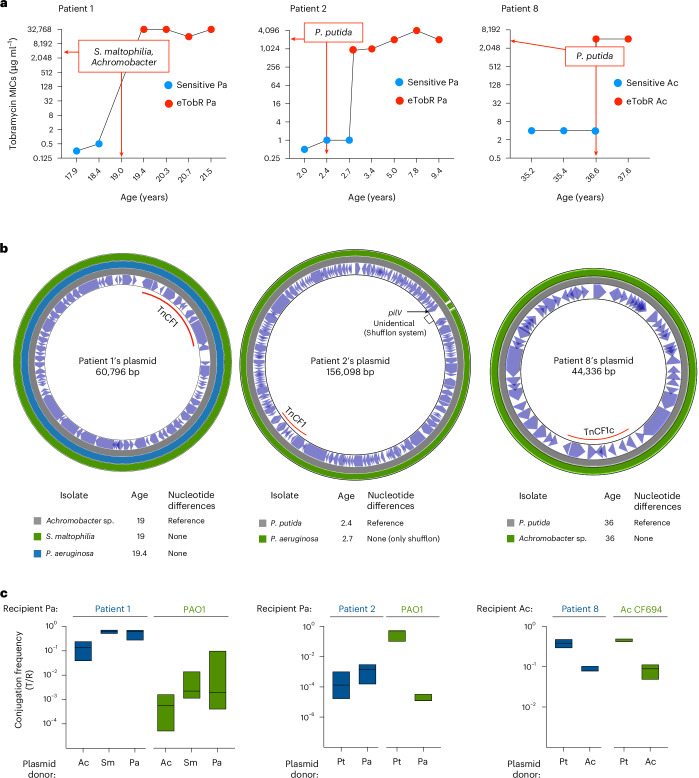
Transfer species appeared before eTobR and can transfer plasmids to sensitive isolates. **a**, Ages (red vertical arrows) at which transfer species (in red boxes; horizontal arrows indicate transfer species Tob MIC values) were detected that contained plasmids acquired by the Pa lineages infecting patients 1 and 2, and the Ac lineage infecting patient 8. **b**, Plasmids detected in the transfer species and the first resistant Pa (patient 1 and 2) and Ac (patient 8) are identical. Plasmid open reading frames are represented in the innermost ring in purple; solid colour outer rings represent plasmids detected in the indicated transfer species and infecting the Pa or Ac lineage. All plasmids were identical within each patient, except in patient 2, where sequences at the end of *pilV* differed, probably due to the presence of a shufflon system^82^. See NCBI GenBank accessions CP199856.1, CP199852.1 and CP199859.1 for plasmids of patient 1; CP199837.1 and CP199842.1 for plasmids of patient 2; and CP199807.1 and CP199811.1 for plasmids of patient 8. **c**, Frequencies at which the transfer species and the Pa and Ac that had acquired eTobR plasmids can transfer eTobR plasmids via conjugation. Plasmid donors were the identified transfer species (Ac and Sm in patient 1 and Pt in patient 2 and 8) and the first-detected plasmid-containing pathogen (‘Pa’ in patients 1 and 2) and Ac (‘Ac’ in patient 8). Recipients were the last-detected plasmid-free Pa (patients 1 and 2) and Ac (patient 8), and PAO1 (patients 1 and 2) and Ac CF694 (patient 8). T, transconjugants, R, recipients. Data are shown as median (range, minimum to maximum) of *n* = 3 independent experiments. See Supplementary Table 14 for validation experiments. Source data

We searched for bacteria that may have carried plasmids into patients’ lungs. Collections from all primary patients (except for patient 3) included other species (Supplementary Table 8). We screened all banked bacteria for the plasmids acquired by the patient’s pathogen by PCR and sequencing, and identified species (called ‘transfer species’) from patients 1, 2 and 8 that carried plasmids 100% identical to those acquired by the pathogen lineages in these patients (Fig. 4a,b). The plasmid acquired by patient 1’s Pa lineage was found in *Achromobacter* sp. and *Stenotrophomonas maltophilia* cultured 5 months before detection in Pa. The plasmid acquired by patient 2’s Pa lineage was found in *Pseudomonas putida* cultured 4 months before detection in Pa. The plasmid acquired by patient 8’s Ac lineage was found in *P. putida* at the same time it was detected in Ac. Notably, these species did not establish chronic infections, as they were only detected once or twice in patient’s cultures (Supplementary Table 8).

We tested the ability of transfer species to transmit their eTobR plasmids to the last-detected sensitive Pa and Ac clones from corresponding patients and found that all could (Fig. 4c, blue bars). They could also transmit their eTobR plasmids to unrelated Pa (PAO1 for patient 1 and 2) and Ac strains (Ac CF694 for patient 8) (Fig. 4c, green bars). Together, these data support the conclusion that the transfer species carried eTobR plasmids to antibiotic-sensitive Pa and Ac inside patients’ lungs.

### eTobR plasmids can transmit between sibling bacteria

Once eTobR emerged, sensitive Pa were consistently detected in only one case (patient 5, Figs. 1 and 2 and Supplementary Table 2), suggesting that the plasmids rapidly disseminated through pathogen populations. This led us to investigate whether Pa and Ac isolates from patients 1, 2 and 8 could transfer acquired eTobR plasmids to plasmid-free sibling bacteria, and we found that all could (Fig. 4c). Sibling-to-sibling plasmid transfer could rapidly spread eTobR within patients and explain plasmid carriage by evolutionarily distinct sublineages within patients (‘*’ in Fig. 3c and Extended Data Fig. 4).

### Plasmids are highly stable ex vivo as they are inside lungs

**Extended Data Fig. 6:**
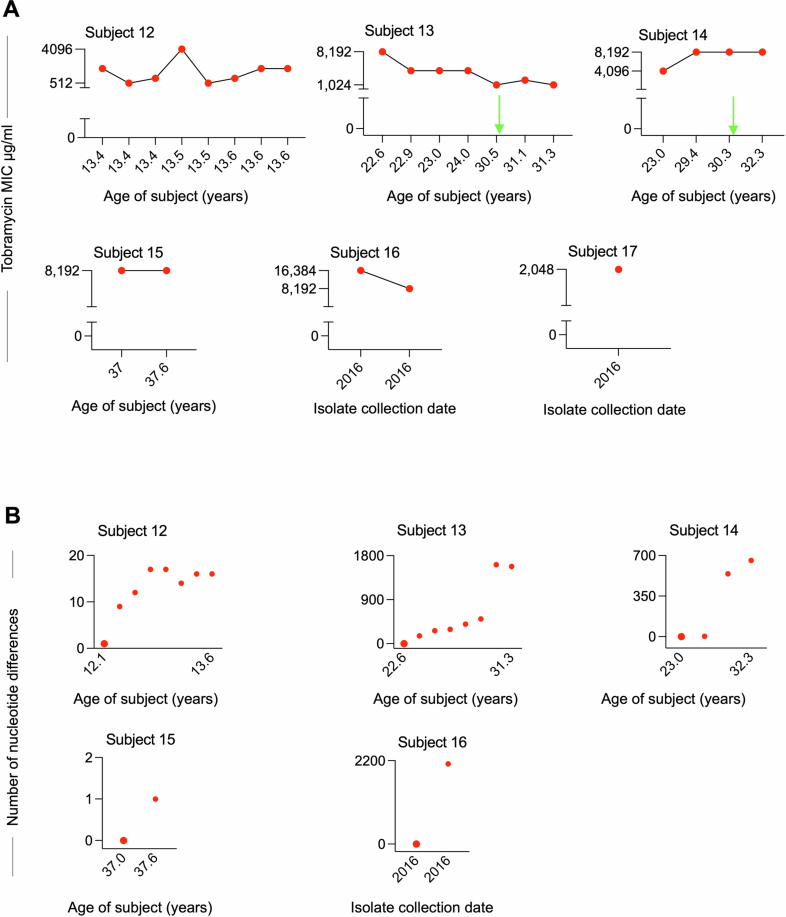
Additional subjects infected with eTobR Pa carrying plasmids with TnCF1. **(A)** Graphs show tobramycin (Tob) inhibitory concentrations (MICs, Y-axes) of longitudinally collected Pa isolates from subjects 12, 13, 14, 15, and 16, and one Pa isolate from subject 17, all from PwCF. X-axes represent subject ages or isolate collection date at non-continuous timepoints. Green arrows indicate when elexacaftor/tezacaftor/ivacaftor (ETI) was initiated in subjects 13 and 14. **(B)** Number of nucleotide differences in whole-genome sequences of isolates from each subject. Sequences of each isolate were compared to the earliest isolate collected from the same subject (indicated by the large red point, used as the reference). Nucleotide differences include single nucleotide variants (SNVs), insertions, and deletions up to 50 nucleotides in length. In all subjects a single, subject-specific Pa strain was present longitudinally at all time points. Source data

In all 8 primary patients, in 3 patients (patients 9–11) without an identified clonally related sensitive isolate (Fig. 1), and in 3 additional patients (patients 12–14, Extended Data Fig. 6), eTobR persisted throughout the entire duration of isolate collection (longest observed duration, 14.1 years). This was notable because inhaled Tob is approved for alternating 28-day ‘on’ and ‘off’ periods^15^, hence Tob selection is intermittent. Furthermore, in patients 10, 13 and 14, eTobR plasmids were either acquired after or maintained during elexacaftor/ivacaftor/tezacaftor (ETI) treatment (green arrows in Fig. 1 and Extended Data Fig. 6a mark ETI initiation). This was notable because ETI treats the basic CF physiological defect^22^, and reduces pathogen density and antibiotic use^23^. These two observations suggest that the plasmids are highly stable, carry low fitness costs, or both.

**Fig. 5:**
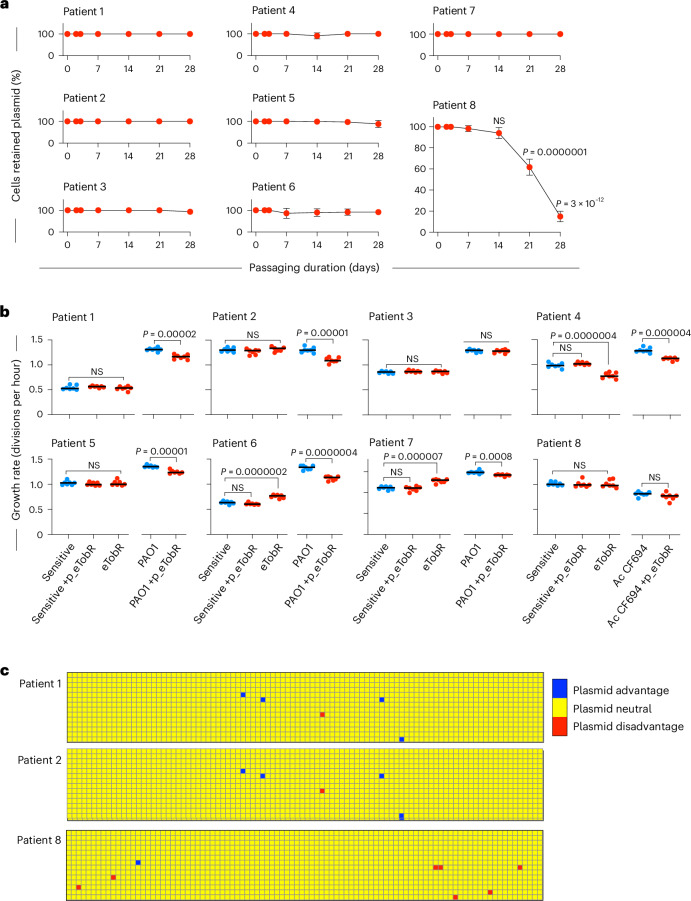
Plasmids are highly stable ex vivo and produce minimal fitness costs. **a**, The first-detected plasmid-containing Pa (patients 1–7) and Ac (patient 8) isolates were serially passaged daily in Tob-free media for 28 days; 96 colonies were tested each day to determine plasmid maintenance. See NCBI GenBank accessions CP199861, CP199842, CP199831, CP199828, CP199822, CP199817, CP199814 and CP199807 for complete plasmid sequences. Data are shown as mean ± s.d. of *n* = 3 independent replicates per timepoint. ****P < 0.0001; NS, not significant; one-way analysis of variance (ANOVA). Source data. **b**, Plasmids produced no measurable growth defect in the Pa or Ac isolates acquiring them. ‘Sensitive’ and ‘Sensitive+p_eTobR’ indicates the last-detected plasmid-free Pa from patients 1–7 or Ac from patient 8 in which the patient-corresponding plasmid had not and had been inserted, respectively; ‘eTobR’ indicates the first-detected plasmid-containing Pa (patients 1–7) and Ac (patient 8); ‘PAO1’ and ‘PAO1+p_eTobR’ indicates strain PAO1 without and with plasmids from patients 1–7, respectively; ‘Ac CF694’ and ‘Ac CF694+p_eTobR’ indicate Ac strain CF694 without and with the plasmid from patient 8, respectively (see Methods). Data are shown as median of *n* = 7 technical replicates; ****P < 0.0001 (one-way ANOVA) and ***P < 0.0008 (unpaired two-tailed Student’s *t*-test). Source data. **c**, Isogenic plasmid-free and plasmid-containing isolates from patients 1, 2 and 8 were tested using phenotypic microarrays across 1,344 growth conditions. Displayed arrays indicate differences in area under the growth curve between plasmid-free and plasmid-containing strains, with each small box representing a different growth condition. Yellow indicates that the plasmid does not affect growth, blue indicates that the plasmid increases growth, and red indicates that the plasmid decreases growth. The experiment was performed twice with consistent results. Individual growth curves are provided in Supplementary Figs. 1–3. Source data

**Extended Data Fig. 7:**
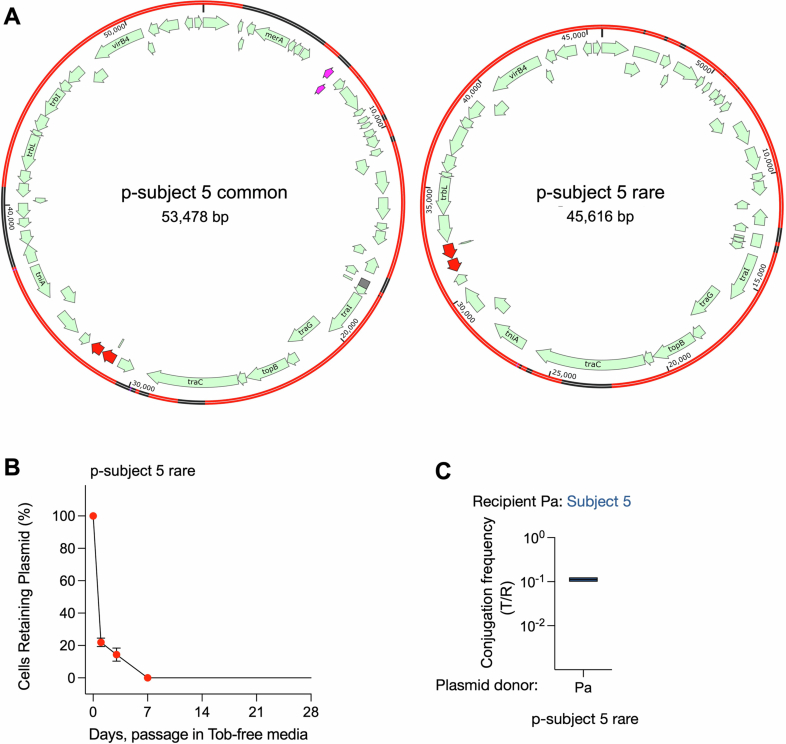
A rare variant of subject 5’s plasmids lacks toxin-antitoxin genes and is unstable but remain conjugative. **(A)** A plasmid variant in subject 5 was found in two isolates. Plasmid maps show the common (53 kb) and the rare (45 kb) plasmids. Red outer regions indicate shared sequences between the two plasmids. Toxin-antitoxin genes (purple) present in the common plasmid are absent in the smaller, rare version. **(B)** Plasmid stability measurements of the rare small plasmid. Pa carrying the plasmid was serially passaged daily in Tob-free media for 28 days with daily 1:2000 back-dilution. Ninety-six colonies were tested at days 0, 1, 3, and 7 to assess plasmid maintenance or loss (see Methods). Data represent mean values from three independent replicates per timepoint; error bars indicate standard deviation. **(C)** The rare small plasmid remains conjugative. Pa carrying the rare plasmid was used as the donor, and the earliest Tob-sensitive Pa isolate from subject 5 served as the recipient. T and R represent transconjugants, and recipient cells, respectively. Data are from three independent conjugation experiments; bars indicate minimum and maximum values, lines indicate the median. Source data

We tested plasmid stability by passaging the first-detected plasmid-containing pathogen isolates in Tob-free medium for 28 days (~650 generations) and found that >95% of bacteria from all patients except Ac from patient 8 continued to harbour plasmids (Fig. 5a). This remarkable stability may be due to the toxin–antitoxin genes found in all studied plasmids (Supplementary Table 9). Such genes enhance maintenance by killing plasmid-free daughter cells^24^. Consistent with this explanation, a plasmid variant from patient 5 that lost *parED* toxin–antitoxin genes was unstable (Extended Data Fig. 7).

### Plasmids impose minimal fitness costs

We inserted plasmids into the Tob-sensitive isolate that preceded plasmid acquisition (called ‘Sensitive’ in Fig. 5b) to generate isogenic plasmid-free and plasmid-containing strains from each patient. This enabled us to study plasmid fitness effects in the clinical isolates acquiring them, before compensatory mutations could arise. Plasmid-free and plasmid-containing isolates had similar exponential growth rates in laboratory medium (compare ‘Sensitive’ and ‘Sensitive+p_eTobR’, Fig. 5b).

Because fitness effects can be condition dependent, we also compared the growth of isogenic plasmid-free and plasmid-containing isolates from patients 1, 2 and 8 in 1,344 nutritional and non-antibiotic stress conditions using phenotypic microarrays^25^. We found no growth differences in 99.6% of these conditions (Fig. 5c and Supplementary Figs. 1–3). Thus, despite conferring extreme resistance, plasmids produced minimal fitness costs in the Pa and Ac lineages that acquired them. The plasmids from 6 of 8 patients did modestly slow growth of the PAO1 reference strain in Luria broth (Fig. 5b), suggesting that fitness effects may be host-strain dependent.

### Plasmids contain a previously undescribed Tn-borne resistance gene

**Fig. 6:**
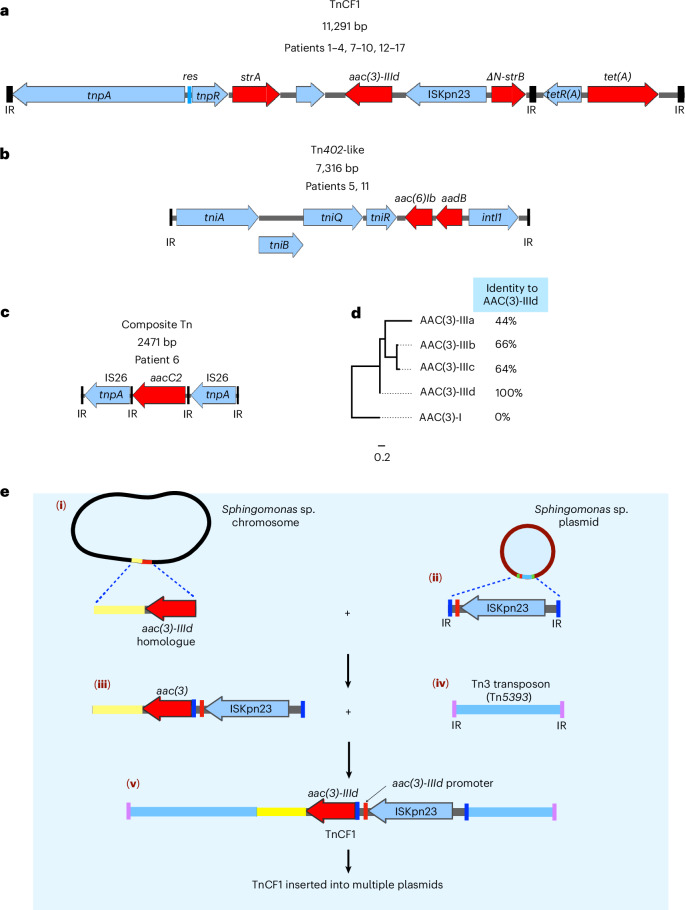
The TnCF1 transposon mediates eTobR in majority of patients. **a**, Map of TnCF1 showing the aminoglycoside-modifying enzyme genes *aac(3)-IIId* (adjacent to the ISKpn23 insertion sequence), *strA*, a truncated ΔN-*strB, tet(A)* (all resistance genes in red) and its 38-bp inverted repeats (IRs) (black). The most common version of TnCF1 is composed of Tn*5393* with *strA*, *aac(3)-IIId*, and *tet(A)* which is present in 11 of 14 patients. *tnpA* is disrupted in TnCF1 from 2 of 14 patients (see Supplementary Table 1 for TnCF1 variants). See NCBI GenBank accession CP199861 for TnCF1 sequence on the plasmid of patient 1. **b**, Pa from patients 5 and 11 carry a Tn*402*-like transposon encoding an aminoglycoside 6-*N*-acetyltransferase (*aac(6)-Ib*) and an aminoglycoside nucleotidyltransferase (*aadB*) on a class 1 integron. See NCBI GenBank accession CP199822 for the sequence of Tn*402*-like transposon on the plasmid of patient 5. **c**, Pa from patient 6 carries a composite transposon (IS26) encoding aminoglycoside 3-*N*-acetyltransferase (*aacC2*). See NCBI GenBank accession CP199817 for the sequence of the composite transposon on the plasmid of patient 6. **d**, Maximum-likelihood tree of AAC(3)-III subtype proteins showing AAC(3)-IIId, rooted on AAC(3)-I. **e**, Model for the origin of TnCF1. (**i**) *Sphingomonas* sp. chromosome sequences contain an *aac(3)-IIId* homologue and contiguous downstream sequences with 83% identity to the *aac(3)-IIId* gene and its contiguous downstream sequence (yellow) on TnCF1. The *aac(3)-IIId* on *Sphingomonas* was not associated with mobilizing elements. (**ii**) A plasmid from *Sphingomonas hankookensis* contained an ISKpn23 sequence and promoter with 100% identity to the ISKpn23 and the *aac(3)-IIId* promoter on TnCF1. (**iii**) We postulate that the ISKpn23 including the promoter jumped to the *Sphingomonas* chromosome adjacent to the *aac(3)-IIId* gene homologue. (**iv**) The ISKpn23 then may have carried the *aac(3)-IIId* gene and its promoter to the Tn3-family transposon Tn*5393*, forming TnCF1. (**v**) TnCF1 was found on many distinct plasmids, including those in transfer species identified here.

**Extended Data Fig. 8:**
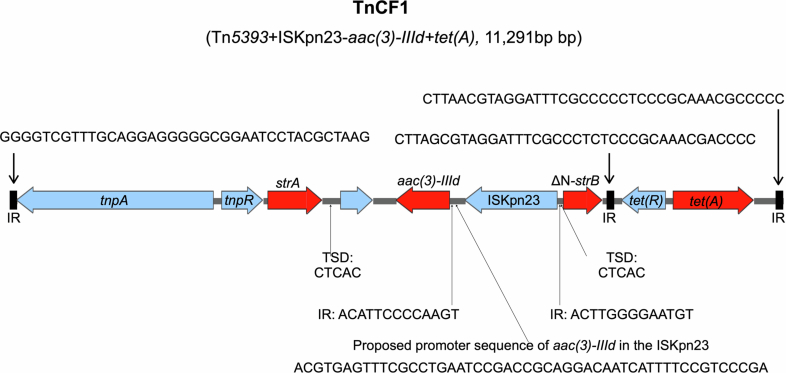
TnCF1 has 38-nucleotide inverted repeats. TnCF1 is a derivative of Tn5393 that has acquired the ISKpn23-*aac(3)-IIId*. In most of the subjects we sequenced, as well as in genome database searches, TnCF1 has ISKpn23-*aac(3)-IIId* and the *tetR(A)*-*tet(A)* genes. TnCF1 carries complete copies of the 81-nucleotide IR present in Tn5393 at the end of the transposon next to *tnpA* and internally at the end of ΔN-*strB*. The sequence at the other end of the transposon next to *tet(A)* shares 38 nucleotides with this 81-nucleotide sequence, suggesting that the conserved 38-nucleotide region (black bars labeled IR) suffices for TnCF1 transposition. The *tnpA-tnpR* region in TnCF1 is nearly identical to that of Tn5393. The sequence and location of the *acc(3)-IIId* promoter (located within ISKpn23), the inverted repeats of ISKpn23, and the target site duplications (TSDs) flanking the fragment introduced by ISKpn23 are also shown.

Plasmids from 13 study patients (patients 1–4, 7–10 and 12–16) and the Pa chromosome from one patient (patient 17) carried variants of a transposon that we termed Transposon CF #1 (TnCF1) due to its prevalence in CF (Figs. 3a, 4b and 6a; see Supplementary Table 1 for TnCF1 variants; see Fig. 6b,c for resistance elements in other patients). TnCF1 encodes a Tn3-family transposase (*tnpA*), a resolvase (*tnpR*) and a transposon recombination site (*res*). It also carries 38-bp inverted repeats and typically generates 5-bp target site duplications in host DNA^26,27^, although 6-bp duplication can occur (Extended Data Fig. 8 and Supplementary Table 10). The sequences of *tnpA*, *res, tnpR* and the inverted repeats of TnCF1 are 99–100% identical to transposon Tn*5393* (ref. ^28^).

**Extended Data Fig. 9:**
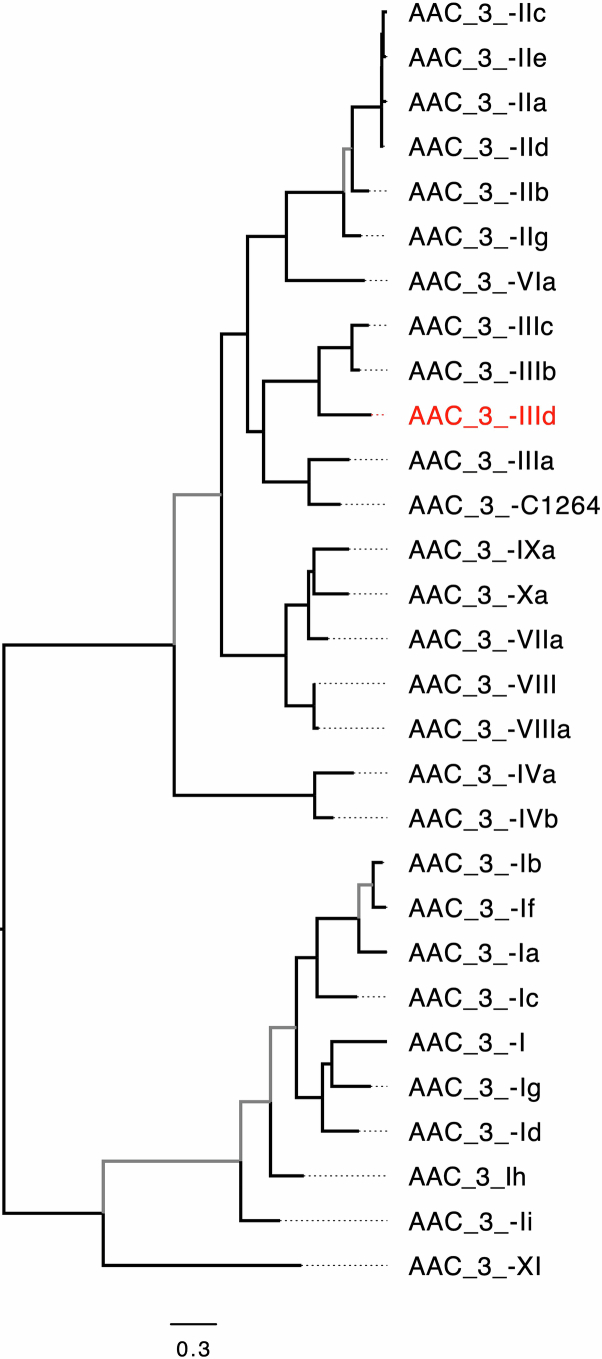
Phylogenetic analysis groups TnCF1’s AAC(3) with AAC(3)-III subtypes. A midpoint-rooted maximum likelihood phylogenetic tree of unique AAC(3) proteins from NCBI reference sequences was constructed using IQ-TREE. Based on this analysis, the AAC(3*)* in TnCF1 was designated AAC(3)-IIId.

TnCF1 contains the *strA* aminoglycoside-modifying gene which is present on Tn*5393* (refs. ^28,29^) and a previously undescribed *aac(3)* aminoglycoside *N*-acetyltransferase (Fig. 6a) not found on Tn*5393*. This *aac(3)* gene was recently discovered using a computational approach that predicts resistance genes (it is labelled *aac(3)-C322* in ref. ^30^, NCBI accession NG_242159). It is not present in the CARD^31^ and ResFinder^32^ resistance gene databases and has not been described as a cause of clinical resistance. Its closest homology is to class IIIb *aac(3*) genes (66% protein identity) which confer Tob resistance^29^ (Fig. 6d and Extended Data Fig. 9). Thus, we designated it as *aac(3)-IIId* (the *c* designation is already in use).

We inserted *strA* and *aac(3)-IIId* with their native promoters individually and in combination into the Pa reference strain PAO1 in single copy and found *aac(3)-IIId* sufficient for eTobR; a single copy increased Tob MICs 1,000-fold (Supplementary Table 11). It also produced resistance to kanamycin and gentamicin. However, amikacin, netilmicin, apramycin and plazomycin were effective against both aminoglycoside-modifying genes carried by TnCF1 (Supplementary Table 11).

### Plasmid-borne TnCF1 is found in pathogens worldwide

We searched sequence databases to identify TnCF1 in other bacteria. A comprehensive search of 9.9 million assembled genomes that included ~43,000 Pa genomes identified 28 unique Pa genomes carrying TnCF1. Most came from people with CF or from an uncharacterized respiratory sample, and 12 were epidemic Pa sequence types^33^ (the Pa from 3 of the 8 primary study patients were also epidemic sequence types). Epidemic sequence types can spread from person to person and account for most clinical Pa infections worldwide^33^.

TnCF1 was present on plasmids in 23 of 28 Pa genomes identified via database searches, and in 18 of 23 cases, the plasmids were highly similar to those identified here (Supplementary Table 12). TnCF1 was also on plasmids in all but 2 of 17 non-Pa genomes where it was identified. Except for 2 *Comamonas jiangduensis* genomes, all genomes containing TnCF1 sequences were human pathogens. These pathogen genomes originated from North and South America, Europe and Asia (Supplementary Table 12). Thus, TnCF1 was almost always plasmid borne, indicating acquisition by horizontal transfer, and it is already present at disparate worldwide locations.

### TnCF1 may have originated from environmental *Sphingomonas*

Identifying the origins of resistance elements is important for devising mitigation strategies. We searched for TnCF1 in NCBI’s metagenomic database and identified it in 24 of 5.2 million samples queried. Notably all but five of these were environmental samples and most contained >1% Pa sequence (Supplementary Table 13). This finding raised the possibility that TnCF1 originated from the environment.

To explore this, we searched NCBI for homologues of the *aac(3)-IIId* gene that were not associated with genetic mobilizing elements, as the absence of such elements implies a species of recent gene origin^34^. This analysis identified a *Sphingomonas* strain from a Chinese environmental sample that contained DNA that was 83% identical to the *aac(3)-IIId* gene and the 983 bp of downstream sequence found on TnCF1 (coloured yellow in Fig. 6e). *Sphingomonas* are intrinsically resistant Gram-negative bacteria ubiquitous in natural and polluted environments^35^. The extent of sequence identity, absence of mobilizing elements and the fact that a long stretch of downstream sequence from the *Sphingomonas* was on TnCF1 suggests this or a related organism as one potential source of *aac(3)-IIId*.

While the *aac(3)-IIId* homologue on this *Sphingomonas* lacked mobilizing elements, the *aac(3)-IIId* on TnCF1 is adjacent to an ISKpn23 insertion sequence (IS). ISKpn23 belongs to the IS1380 family, which is known to mobilize adjacent DNA without a second flanking element^36^. We searched NCBI using TnCF1’s ISKpn23 and identified a plasmid in a *Sphingomonas* strain from wastewater in Hong Kong containing DNA that was 100% identical to TnCF1’s ISKpn23 and included the *aac(3)-IIId* promoter on TnCF1 (also 100% identical) (Fig. 6e). Finding the identical ISKpn23 in the same bacterial species (*Sphingomonas*), along with ISKpn23’s known ability to mobilize genes suggests a hypothetical model wherein plasmid-borne ISKpn23 could have jumped to the chromosome of *Sphingomonas*, landing adjacent to the *aac(3)-IIId* homologue. The ISKpn23 could have then carried *aac(3)-IIId* to a Tn3-family (Tn*5393*) transposon to form TnCF1. Consistent with the high dissemination capacity of Tn3-family transposons^27^, TnCF1 was then acquired by multiple plasmids. The 13 plasmid sequences we completed containing TnCF1 included 5 unique types (Supplementary Table 7).

## Discussion

Here we describe multiple independent cases from disparate locations in North America wherein antibiotic-sensitive pathogens that had established chronic lung infections suddenly developed ~10,000-fold increases in Tob resistance after exogenous genes were transferred to them. The following evidence supports the conclusion that these pathogens acquired resistance genes while they were inside human lungs:

1. In all primary patients, pre-existing sensitive and eTobR isolates were clonally related. Thus, the sudden emergence of eTobR was not a consequence of strain displacement.
2. All pre-existing sensitive isolates lacked plasmids carrying resistance genes, whereas succeeding eTobR isolates had acquired plasmids carrying eTobR genes. Experimental transfer of these plasmids to patients’ sensitive isolates produced the eTobR phenotype.
3. In all patients where the ancestry of the Pa isolates was clear, isolates that had acquired eTobR plasmids were descendants of sensitive isolates that had been collected earlier. This finding and the remarkable stability of the plasmids is inconsistent with the alternative mechanism wherein plasmid-containing isolates were acquired first.
4. In three patients we identified the bacteria that probably carried eTobR plasmids inside the patient. These likely transfer species carried genetically identical plasmids, and they readily transferred plasmids to sensitive pathogen isolates ex vivo.

Together these findings support the conclusion that previously sensitive pathogens acquired genes causing extreme resistance inside these patients.

Gene acquisition is a key driver of bacterial evolution^37^. A recent study demonstrated its importance on a global scale by analysing thousands of Pa strains collected over a century from locations worldwide^33^. This work showed that over long timescales, certain Pa sequence types acquire genes that enhance their capacity to cause human infections^33^. Our study adds to emerging evidence demonstrating gene acquisition on a more granular scale, that is, in bacteria already inside patients. For example, previous work identified 5 patients in whom identical carbapenemase-encoding plasmids were carried by multiple intestinal bacteria, suggesting that within-patient plasmid transfer occurred^38^. Other publications report individual cases wherein pathogens appeared to acquire resistance plasmids in vivo^39–45^. These data and our findings raise the possibility that pathogens in established infections may frequently acquire exogenous genes that enable them to adapt to conditions in vivo (see below).

Where did the TnCF1 transposon come from? Understanding resistance gene origins could help predict and counter resistance. Our work on TnCF1 that caused eTobR in 14 of 17 cases identified here suggests that it may have been created when an IS captured the *aac(3)-IIId* gene from the chromosome of an environmental organism. The IS, its promoter and the *aac(3)-IIId* gene may have then jumped to a Tn3-family transposon (Tn*5393*) to produce TnCF1. Characteristics of the particular IS (ISKpn23) and transposon type (Tn3-family) involved may have been critical. ISKpn23 has the unusual capacity to mobilize adjacent DNA when present in single copies^36,46^. Tn3-family transposons are notorious for disseminating resistance genes in part due to their transposase, which uses a copy-paste mechanism and thus creates two Tn copies during transposition^27^. One is transferred, the other is retained, and both can transpose again. Consistent with this propensity for dissemination, we found that TnCF1 had been acquired by multiple different plasmids in our patients and in genomic databases.

In 3 cases, we identified the bacterial species (*P. putida*, Ac and *S. maltophilia*) that probably carried plasmid-borne TnCF1 into the patients’ lungs. Their capacity to grow in the environment and infect humans may be a key factor in their ability to serve as plasmid transfer species. It was interesting that the likely transfer species only transiently infected patients. We speculate that before plasmid transfer, high-dose Tob treatment may have prevented established sensitive pathogens from saturating the available lung growth environments. However, once resident pathogens acquired eTobR, the transfer species may have been outcompeted by the established pathogen as transfer species are probably less capable of infecting lungs than canonical pathogens such as Pa^47^. The possibility that the vacant niche exploited by transfer species was fleeting could also explain why the isolate collections did not capture them in the other patients we studied.

How widespread is TnCF1-mediated resistance? Our analysis of sequence databases found TnCF1 in pathogens from 15 countries across 3 continents, even though it has not been recognized as a cause of clinical resistance. This finding parallels earlier reports on emerging resistance elements. For example, the sequences of the plasmid-borne colistin resistance gene *mrc-1* had been deposited in databases by investigators from 16 countries across two continents before being recognized as cause of clinical resistance in 2015^48,49^. Likewise, the element carrying the NDM-1 metallo-β-lactamase was discovered in 2008, and by 2010 had been identified in Asia, Europe, Australia and North America^50^. Thus, widespread dissemination of these elements can occur before they are clinically recognized. TnCF1’s ability to confer potent, stable and low-cost resistance, and its acquisition by epidemic Pa sequence types could all contribute to its wide dissemination. Surveillance of sequence data using tools that can predict resistance elements, and analysis of clinical isolates could improve detection and trigger control measures.

Why was resistance so extreme and durable? The *aac(3)-IIId* gene is a class III *aac(3)*, and class III *aac(3)* genes are only found in 0.1% of Pa^51^. Future work is needed to determine whether this gene has particular potency, if the ISKpn23-transferred promoter is particularly strong, or both. Our preliminary analysis using the *aac(3)-IIIa*^52^ structure identified 20 positions that may bind to aminoglycosides, and *aac(3)-IIId* has unique residues at 10 of these. These or other differences could account for its potency.

After it emerged, eTobR was continuously present in all cases we studied (longest: 14.1 years). This was notable because antibiotic selection was probably only intermittent. Two factors could contribute to this durability. First, all identified plasmids contained addiction systems that inhibit daughter cells that loose plasmids during division. The importance of these systems was illustrated by the natural experiment in patient 5, where a variant without an addiction system was highly unstable.

Second, eTobR plasmids produced minimal fitness costs in laboratory medium and in nearly all 1,344 stress conditions we tested. This was notable as the plasmids have characteristics associated with reduced fitness, including large sizes^53^, extreme resistance^54^ and transfer from a different species^55^. Moreover, high plasmid fitness is often due to compensatory evolution of the plasmid or host genome^53^. This mechanism is unlikely here, as our fitness experiments used the first plasmid version detected in each patient, inserted into sensitive Pa or Ac collected before plasmids were acquired. These findings suggest that low fitness costs are probably an inherent property of these eTobR plasmids.

This study had limitations. While the bacterial collections used were extensive and contained pathogens and co-infecting species, only 1–3 isolates of each species were generally collected at each timepoint. Thus, we are unable to determine the dynamics of plasmid uptake. Likewise, it is possible that the transfer species were present at low abundances for longer times than reflected by culture results, or that species undetected in clinical cultures participated in plasmid transfer. These could include transmissible Pa strains or bacteria endogenous to the host. Limitations in comprehensively sampling bacteria present in humans make it impossible to rule out these possibilities. In addition, it is possible that the plasmids carried fitness costs or conferred benefits in addition to eTobR within patients’ lungs. The phenotypic microarray experiments investigated potential positive or negative fitness effects in >1,000 conditions, but do not model all in vivo conditions.

The high homology between TnCF1 and sequences from the identified *Sphingomonas* (including the *aac(3)-IIId* homologue + downstream sequences without associated mobilizing elements) and *Sphingomonas* plasmid (including ISKpn23 + its promoter) suggests that TnCF1 could have originated from *Sphingomonas*. However, we could not identify intermediary species or elements through which TnCF1 or its components may have passed before being acquired by transfer species. Moreover, the bias of sequence databases towards host-associated organisms^56^ raises the possibility that TnCF1 may have also originated in other species not yet represented. In addition, this study focused on Tob resistance in Pa-infected patients with chronic CF lung infections. Future work could determine whether similar mechanisms operate in other conditions. Finally, available clinical data do not enable us to determine whether the emergence of eTobR was associated with changes in clinical parameters such as lung function or disease flares.

Our findings have implications for infection treatment and pathogen adaptation. While any cause of resistance could compromise treatment, the mechanism identified here could be particularly consequential. The magnitude of resistance produced will probably overcome any dosing regimen possible in humans, including the high levels achieved by inhalation in lungs, topically in wounds, and by drug-impregnated medical devices. The instantaneous onset of resistance after treatment was started could obviate the predictive value of antibiotic-sensitivity testing (usually done when infection is first detected) and prevent prescription changes that aim to limit resistance. The high-stability and low fitness costs of the genes that were acquired here will probably make drug holidays ineffective, as was seen in our patients. In addition, existing infection control measures may be inadequate. Even transient infection with organisms such as *P. putida* that are generally considered medically unremarkable can transfer genes that transform a pathogen’s ability to resist treatment.

Gene transfer from transiently infecting bacteria could also enable pathogens inside human organs to adapt to conditions beyond antibiotics, as environmental sites contain genes that could increase pathogenicity in innumerable ways^57^. In the people studied here, requisite gene phenotypes included eTobR, facile transmissibility to Pa and Ac, and durability during antibiotic-free periods. We identified multiple cases around North America where such genes were transferred to pathogens inside human lungs. This same mechanism could transfer other genes, including those that counter limitations in in vivo nutritional availability, host immune activity, or existing or novel therapeutics. Mitigation strategies involving environmental monitoring and containment, and new strategies to compromise in vivo gene transfer could be developed to counter this threat.

## Methods

### Study patients

We screened longitudinal tobramycin susceptibility profiles of isolates from 3 sources including isolates from (1) the Singh Laboratory at the University of Washington, (2) the Cystic Fibrosis Microbiology Outcomes Advancement Core (MOAC) at the Seattle Children’s and the University of Washington and (3) the Calgary Adult Cystic Fibrosis Clinic Biobank (CACFB). In addition, we requested Pa isolates from other biobanks and laboratories after we found isolates carrying TnCF1 in sequences deposited at NCBI (see Supplementary Tables 1 and 2 for details about the isolates). Local ethics and consent procedures were followed at each site providing isolates and all isolates were de-identified from protected health information. patients 1–7 and 9–17 were chronically infected with Pa and patient 8 with Ac, and all had at least two sequential Tob-sensitive isolates banked that preceded the appearance of isolates with Tob MICs of at least 1,024 µg ml^−1^. Primary study patients (patients 1–8) had at least two subsequent eTobR isolates clonally related to the initial sensitive isolate collected at different timepoints. The sources of each isolate and MICs are listed in Supplementary Table 2. Isolates from MOAC, CACFB and other sources were cultured by the clinical microbiology laboratories at each site, and isolates from the Singh Laboratory were cultured as described in ref. ^58^.

### Antibiotic susceptibility and mutation frequency measurements

Minimum inhibitory concentrations were measured using the Clinical and Laboratory Standards Institute (CLSI) method^14^. Bacteria from freezer stocks were grown overnight in Luria–Bertani (LB) broth at 37 °C with 220 r.p.m. shaking. Cultures were then diluted to a concentration of 10^6^ cells per ml in cation-adjusted Mueller–Hinton Broth (caMHB) and exposed to a range of antibiotic (Research Products International) concentrations for 24 h with gentle shaking. The MIC was defined as the lowest antibiotic concentration that completely inhibited bacterial growth (turbidity).

To measure mutation frequencies of lineages, the earliest available isolates (Tob-sensitive) from patients 1–8 (and PAO1 (ref. ^59^) as a control) were grown overnight in LB broth, and 10^8^ cells were plated on LB agar with 300 µg ml^−1^ rifampicin in triplicate^60^. The plates were incubated at 37 °C for 48 h, and the number of colony-forming units (c.f.u.s) was counted.

### Genome sequencing

Libraries for Illumina sequencing were prepared using the Illumina DNA Prep kit as described in the Illumina reference guide, from genomic DNA extracted using the QIAcube instrument with DNeasy Blood & Tissue kits (Qiagen). Sequencing was performed on the Illumina NextSeq 550 System instrument at UW’s CF Genomics Core facility. Sequencing libraries for Oxford Nanopore long-read sequencing were prepared using the Oxford Nanopore ligation sequencing kit (SQK-LSK114) from DNA extracted using the Nanobind CBB kit (PacBio). Sequencing was performed using GridION 10.4.1 flow cells (FLO-MIN114) by Plasmidsaurus.

### Bioinformatics analysis

#### Sequence read preprocessing

Before assembly and alignment, Illumina reads were filtered for duplicates, sequence adapters, base quality and read length using the HTStream 1.3.3 pipeline (https://s4hts.github.io/HTStream/). Briefly, defaults were used for hts_SuperDeduper, hts_SeqScreener and hts_AdapterTrimmer, 20-bp windows with a minimum quality score of 10 was used for hts_QWindowTrim, and half the mean read length was used as the minimum read length for hts_LengthFilter. Oxford Nanopore long reads were downsampled to 100× coverage with FiltLong 0.2.1 (https://github.com/rrwick/Filtlong), with a minimum 1 kb length requirement.

#### Genome assemblies and annotations

For Illumina short-read assemblies, SPAdes 4.0.0 with default settings was used to generate whole- genome contigs^61^. For long-read assemblies, Trycycler 0.5.5 was used to complete the genomes using a hybrid assembly process with Illumina short reads and Oxford Nanopore long-read sequences, using the methodology described in the Trycycler wiki (https://github.com/rrwick/Trycycler/wiki/Illustrated-pipeline-overview)^62^. Bakta 1.9.4 was used to annotate all assemblies^63^. Kraken 2 was used to identify bacterial genera and species^64^. Whole-genome sequencing was used to determine multilocus sequence typing (MLST) of the isolates^65^. Types of mobile element were identified with ISfinder^26^.

#### Sequence read alignment, variant calling and sequence comparison

Illumina reads were aligned to reference sequences using bwa-mem2 (2.2.1)^66^. LoFreq 2.1.5 was used for variant calling in chromosomes and plasmids^67^. Briefly, for each patient, the genome of the earliest available isolate was completed and used as a reference. All isolates were whole-genome sequenced, and sequence reads were mapped to the patient’s reference genome. Nucleotide differences with a LoFreq quality value of at least 500, frequency of at least 90%, and sequencing coverage of ≥10× (mean coverage 81×) were retained for further analysis. Nucleotide differences include single-nucleotide variants (SNVs), insertions and deletions up to 50 nucleotides in length. The same methods were used to call nucleotide differences in the plasmids, with the earliest complete circular plasmid used as a reference. We used this approach to determine whether the plasmid sequences are identical in the transfer species and in Pa (patients 1 and 2) and Ac (patient 8), because the Trycycler 0.5.5 tool used to complete plasmid sequences in NCBI was unable to confirm plasmid identity. Homology and sequence identity between ISKpn23–*aac(*3)*-IIId* and the *Sphingomonas* sp. chromosome sequences (NZ_JAPCIA000000000 and NZ_JBCNMA000000000), as well as the *Sphingomonas hankookensis* plasmid (CP117026.1), were assessed using BLASTN. For plasmid typing and annotation, MOB-suite 3.1.9, Bakta 1.9.4, oriTfinder2 and PlasAnn (https://plasann.rochester.edu/analysis) were used^63,68,69^. Toxin–antitoxin genes were determined using TADB (3.0)^70^.

#### Phylogenetic analysis

The phylogenetic trees were generated using IQ-TREE 2.3.6 software^71^. Briefly, we first searched for a heterologous *P. aeruginosa* strain (*Achromobacter* sp. for patient 8) from the NCBI GenBank database to be used as an outgroup and to root the tree on it. LoFreq was used to find and extract single-nucleotide variants in the isolates of each patient, including the heterologous strain. The early complete genome was used as a reference for each patient. The variant sequences were used as input into the IQ-TREE software to generate maximum-likelihood phylogenetic trees. Using the IQ-TREE ModelFinder, we chose the TVM + F + ASC model for all trees, with the heterologous *P. aeruginosa* serving as the outgroup. The trees were visualized in FigTree 1.4.4 (https://github.com/rambaut/figtree), rooted on the heterologous Pa or Ac. Heterologous Pa isolates SRR14628342, SRR2939362, SRR1014444, ERR953487, SRR1014306, SRR2939340 and SRR14790817 from NCBI were used for tree rooting for patients 1, 2, 3, 4, 5, 6 and 7, respectively. Heterologous Ac isolate SRR2821369 from NCBI was used for tree rooting for patient 8. After the tree was generated, the tips of the branches were annotated with the age of the patient in years (time of isolate collection). For the *aac(3)-III* subtype tree, we used the same approach as described above. A maximum-likelihood tree was inferred using NCBI reference protein sequences for *aac(3)-III* subtypes and *aac(3)-I*, with rooting on *aac(3)-I*. BLASTP was used to calculate sequence identity to AAC(3)-IIId.

#### TnCF1 search in genomic and metagenomic databases

We performed searches for TnCF1 in ~9.9 million publicly available assembled genomes within the WGS, Microbe and RefSeq databases using the NCBI Pebblescout tool^72^; and nucleotide (nt) and RefSeq databases using the NCBI Blast web tool^73^. Nearly 43,000 of the assembled genomes were Pa. In all assembled genome searches, ISKpn23–*aac(3)-IIId* appeared as a single unit.

We also searched ~5.22 million metagenomic samples from the NCBI Sequence Read Archive using the *K*-mer search engine of the Logan Contigs database^74^ and NCBI Pebblescout. Further, we downloaded a subset of the 621,236 WGS Metagenomic Logan Contigs based on partial matches for further analysis using direct alignments with the MMseqs2 software^75^. We classified the identified ISKpn23–*aac(3)-IIId* as part of TnCF1 when at least two inverted repeats, *strA* and ΔN-*strB* genes associated with Tn*5393* were also present.

#### Plasmid conjugation and transformation

Rifampicin-resistant Pa and Ac clinical strains used as recipients in conjugation experiments were generated by growth on LB plates with 300 µg ml^−1^ rifampicin, with stability of the rifampicin-resistant phenotype confirmed by two subsequent subcultures on LB plates containing rifampicin. For conjugation, donor and recipient strains were grown overnight in LB. Cells were then washed 3 times in PBS, and 10^9^ donor and recipient cells were applied to nitrocellulose paper on LB agar, incubated for 20 h, serially diluted and plated on LB agar with 50 µg ml^−1^ rifampicin, 256 µg ml^−1^ Tob, or both and grown for 24 h at 37 °C. Conjugation frequencies were calculated by dividing the total number of transconjugants by the number of recipient cells (T/R)^76^. Recipient strains were rifampicin-resistant, plasmid-free clinical Pa and PAO1 for patients 1 and 2, and rifampicin-resistant, plasmid-free clinical Ac and Ac CF694 for patient 8. Donors included Ac, Sm and eTobR Pa (patient 1), Pt and eTobR Pa (patient 2), and Pt and eTobR Ac (patient 8).

Transconjugants were confirmed by qPCR, colony morphology and *rpoB* sequencing. qPCR used probes specific for recipient Pa (*gyrB*, patients 1 and 2), recipient Ac (*rpoB*, patient 8) and plasmid-borne *aac(3)-IIId*. Transconjugants tested positive for both recipient and plasmid probes, whereas recipients lacked *aac(3)-IIId* and donors lacked *gyrB/rpoB*. Colony morphology further distinguished donor, recipient and transconjugant strains on selective media. For Pa-to-Pa (patients 1 and 2) and Ac-to-Ac (patient 8) conjugations, transconjugants could not be definitively confirmed by *gyrB/rpoB* qPCR or colony morphology alone. These were therefore validated by sequencing the *rpoB* gene to detect the mutation present only in rifampicin-resistant recipients, and qPCR for *aac(3)-IIId*. Recipients carried the *rpoB* mutation, whereas donors did not. Details of the transconjugant confirmations are listed in Supplementary Table 14.

For transformations, donor bacteria were grown overnight in LB broth with 16 µg ml^−1^ Tob. Plasmids were extracted using the ZymoPURE kit, following the low copy number protocol (Zymo). Recipient bacteria were prepared by washing overnight LB cultures twice with 0.3 M sucrose. Recipient bacteria and plasmid were mixed and electroporated. The cell mixture was then incubated for 1 h at 37 °C with shaking and plated on LB with 256 µg ml^−1^ Tob.

#### Inserting *aac(3)-IIId* and *strA* onto the chromosome of PAO1

Copies of *strA*, *aac(3)-IIId* or both genes along with their promoters (as predicted by BPROM, www.softberry.com/berry) were inserted into the chromosome of PAO1 at the Tn7 site as described in ref. ^77^. Briefly, *strA* and *aac(3)-IIId* and 40 bp upstream of their predicted promoters were PCR amplified using primers flanked by adaptors for the pUC18-mini-Tn7T-Gm vector (Addgene, 63120) and assembled into NsiI/SacI-digested pUC18-mini-Tn7T-Gm using the Gibson assembly approach (New England Biolabs). This vector and the helper plasmid pTNS2 (Addgene, 64968) were electroporated into PAO1, and colonies with integrated resistance genes were selected on LB plates with 60 µg ml^−1^ gentamicin. The pFLP2 plasmid was then introduced by electroporation to remove the gentamicin marker, and the plasmid was resolved by growth on yeast-tryptone medium supplemented with 10% sucrose.

#### Plasmid detection and copy number measurements

Multiplexed qPCR was used to screen co-infecting bacteria (that is, species detected in patient’s cultures other than chronically infecting Pa and Ac lineages) for the presence of the transferred plasmids in all primary patients. Briefly, probes targeting 16S rRNA, *gyrB* (for Pa) or *rpoB* (for Ac), and *aac(3)-IIId* (*aac(6)-Ib* for patient 5 and *aacC2* for patient 6) were added to qPCR reactions containing genomic DNA template and Luna universal qPCR master mix (New England Biolabs), and run on a Bio-Rad CFX96 Real-Time PCR system (Bio-Rad). Primers and probes were obtained from Integrated DNA Technologies and are listed in Supplementary Table 15. Species identities of co-infecting bacteria were defined by the clinical laboratories at MOAC and CACFB, and confirmed by 16S sequencing as in refs. ^78,79^. In addition, conventional PCR was used to screen and detect the plasmid backbones. In patient 1’s plasmid, a conserved gene downstream of *parA* was used, while in patient 2’s plasmid, the *dotB* gene was used.

Droplet digital PCR (ddPCR) copy number measurements used probes targeting a single-copy chromosomal gene *gyrB* for Pa and *rpoB* for Ac labelled with FAM, and a gene on the plasmid labelled with HEX (Supplementary Table 15). Three isolates per patient were grown overnight in LB with 16 µg ml^−1^ Tob and ddPCR performed using ~100, ~500 and ~2,500 cells per reaction using the ddPCR Multiplex Supermix (Bio-Rad) on the Bio-Rad QX200 system. Plasmid copy number was calculated using Bio-Rad’s Quantasoft software. Primers and probes used in this study are listed in Supplementary Table 15.

#### Measuring plasmid fitness costs

The fitness cost of plasmid carriage was determined using three pairs of isolates in all 8 patients. These include (1) temporally consecutive and clonally related plasmid-free (‘Sensitive’) (that is, the last Tob-sensitive) and eTobR plasmid-carrying transconjugants (‘Sensitive + p_eTobR’) isolates. Pa and Ac plasmid-free isolates are denoted by ‘*’, and plasmid donor eTobR isolates denoted by ‘#’ in Supplementary Table 2. (2) The first detected plasmid-carrying eTobR Pa and Ac isolates (‘eTobR’). Isolates are denoted by ‘#’ in Supplementary Table 2; (3) the *P. aeruginosa* PAO1 laboratory strain (for patients 1–7), and in *Achromobacter* sp. CF694 in which each patient’s plasmids had not (‘PAO1’ or ‘Ac CF694’) or had (‘PAO1 + p_eTobR’ or ‘Ac CF694 + p_eTobR’) been inserted. The Tob-sensitive Ac CF694 was isolated from a CF patient by the Singh Laboratory. Strains were grown overnight in LB, diluted to an optical density at 600 nm (OD_600_) of 0.15, incubated in 96-well plates with shaking at 37 °C for 24 h, with OD_600_ measurements taken every 5 min using a Synergy H1 Hybrid Reader (BioTek). Growth rate was determined as the maximum slope of the exponential phase, calculated using the easy linear method^80,81^.

Sensitive isolates from patients 1, 2 and 8 (denoted by the symbol ‘*’ in Supplementary Table 2) with and without their strain-specific plasmids, were studied using Biolog Phenotypic Microarrays (Biolog) as described in ref. ^25^. Microarray plates used included PM1-2 (measures carbon utilization), PM9 (measures osmotic/ionic response), PM10 (pH sensitivity) and PM11-20 (chemical sensitivity). In total, these plates tested 1,344 conditions. Biolog File Management/Kinetic Analysis and Parametric Analysis software programs were used to display respiration (growth) curves and to calculate the area under the curve (AUC) for each well. A plasmid was considered to have an effect if the AUC value changed from the beginning of the experiment and continued to show a consistent effect through the 48-h incubation period. A plasmid effect on growth was assigned on the basis of visual analysis of the pairwise growth curve overlays, looking for differences beginning within 24 h of growth (Supplementary Figs. 1–3).

### Reporting summary

Further information on research design is available in the Nature Portfolio Reporting Summary linked to this article.

## Supplementary information

Supplementary Information Supplementary Figs. 1–3.

Reporting Summary

**Supplementary Table 1** Study patients in whom TobR emerged in vivo, **Supplementary Table 2** List of clinical isolates used in this study (*N* = 277), **Supplementary Table 3** Chromosomal content comparison between Tob-sensitive and eTobR isolate pairs, **Supplementary Table 4** Nucleotide substitution rates for isolates from patients 1–8, **Supplementary Table 5** Mutations in genes implicated in Tob resistance in consecutive sensitive and eTobR isolate pairs, **Supplementary Table 6** Consecutive sensitive and eTobR isolate pairs only have few nucleotide differences (ND), **Supplementary Table 7** Classification of plasmids detected in the 11 patients infected with eTobR isolates, **Supplementary Table 8** Detected co-infecting bacteria in the 8 primary patients, **Supplementary Table 9** Toxin–antitoxin genes encode in the plasmids of isolates from patients 1–8, **Supplementary Table 10** Target site duplication sequences identified after mobilization of TnCF1 in host DNA, **Supplementary Table 11** MICs of PAO1 carrying single copies of *aac(3)-IIId*, *strA*, both genes, or the plasmid-containing TnCF1 from patient 1, **Supplementary Table 12** TnCF1 detection in assembled genome databases, **Supplementary Table 13** TnCF1 detection in metagenomic databases, **Supplementary Table 14** Transconjugant confirmation by qPCR and sequencing, **Supplementary Table 15** List of primers and probes used in this study.

## Source data

**Source Data Fig. 1** Isolates from patients 1–11, with the age (in years) of each patient at the time of isolation and the corresponding tobramycin MIC values, **Source Data Fig. 2** Isolates from patients 1–11, with patient age (in years) at the time of isolation listed for each isolate, along with the number of nucleotide differences relative to the first available isolate from each patient, **Source Data Fig. 3b** Tobramycin MICs for sensitive clinical isolates from each patient, and the corresponding MICs after in vitro plasmid insertion, **Source Data Fig. 4c** Conjugation frequencies of plasmid transfer from donor species, Sm, Ac, and Pt strains, and Pa/Ac that had acquired eTobR plasmids, to susceptible recipient isolates from patients 1, 2 and 8, as well as laboratory strains PAO1 and Ac CF694, **Source Data Fig. 5a** Plasmid stability in tobramycin-free media over 28 days. The first detected plasmid-containing Pa (patients 1–7) and Ac (patient 8) isolates were serially passaged daily, and the percentage of isolates carrying the plasmids is shown, **Source Data Fig. 5b** Growth rates of isogenic plasmid-free and plasmid-carrying strains, as well as clinical eTobR isolates, **Source Extended data Fig. 1a** Isolates from patients 1–8, with patient age (in years) at the time of isolation listed for each isolate, along with the number of nucleotide differences relative to the first available isolate from each patient, **Source Extended data Fig. 1b** Number of rifampicin-resistant mutants growing on high rifampicin concentrations, used to assess a hypermutator phenotype in isolates from each patient, **Source Extended data Fig. 3a** Plasmid copy number in plasmid-containing Pa (patients 1–7) and Ac (patient 8) isolates, **Source Extended data Fig. 3b** Tobramycin MICs of sensitive PAO1 and Ac CF964 strains following in vitro plasmid insertion, **Source Extended data Fig. 6a** Isolates from patients 12–17, with patient age (in years) at the time of isolation listed for each isolate, along with the corresponding tobramycin MIC values, **Source Extended data Fig. 6b** Isolates from patients 12–17, with patient age (in years) at the time of isolation listed for each isolate, along with the number of nucleotide differences relative to each patient’s first available isolate, **Source Extended data Fig. 7b** Plasmid stability measurements of the rare small plasmid, **Source Extended data Fig. 7c** Conjugation frequency of rare plasmid

## Data Availability

All sequence data generated in this study can be obtained using accession and other reference numbers provided in Supplementary Table 2 and are listed in NCBI under BioProject PRJNA1280179. The *Stenotrophomonas maltophilia* (patient 1) and *Pseudomonas putida* (patient 2) designations do not match the NCBI species calls; however, the species names used in this study follow standard medical microbiology laboratory identifications based on mass spectrometry. Short-read data for eight isolates from patients 7 and 15, previously sequenced by the Harrison Laboratory (University of Pittsburgh) and the reads used in this study, are listed with their SRA run numbers in Supplementary Table 2. The sources for obtaining the isolates used in this study are provided in Supplementary Table 2. Source data are provided with this paper.
