# Supplementary material for: Within-patient gene transfer between transiently and chronically infecting bacteria causes extreme antibiotic resistance during lung infections
Source: Nat Microbiol. 2026 Jul 23;11(8):2321–35. doi: 10.1038/s41564-026-02414-3 (PMC13423793; doi:10.1038/s41564-026-02414-3)
Supplement: Supplementary file 1 — Supplementary Figs. 1–3. [file 41564_2026_2414_MOESM1_ESM.pdf]

nature microbiology

Supplementary information https://doi.org/10.1038/s41564-026-02414-3

Within-patient gene transfer between transiently and chronically infecting bacteria causes extreme antibiotic resistance during lung infections

In the format provided by the authors and unedited

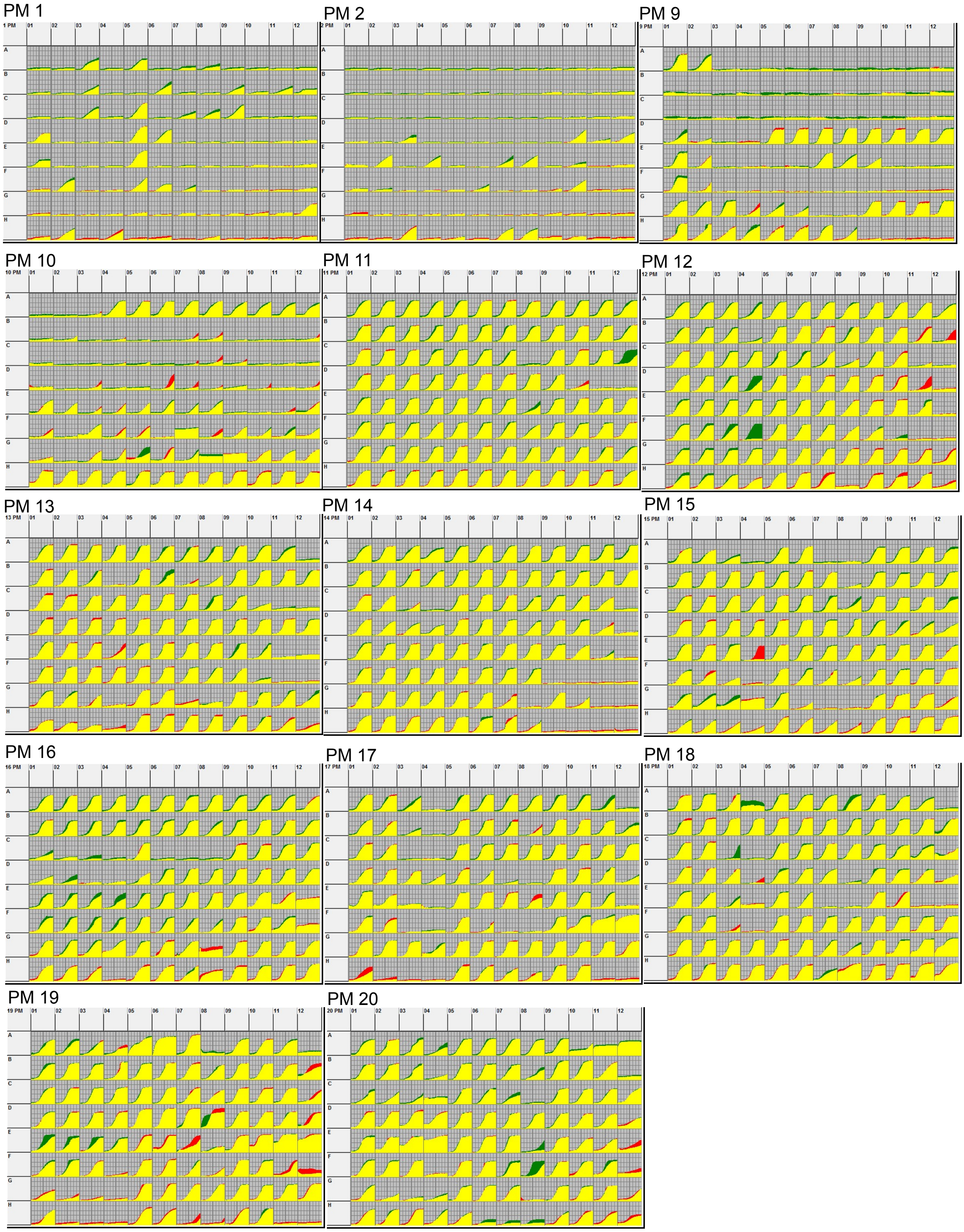

Supplementary Fig. 1. Phenotypic microarrays experiments show plasmid from subject 1 confer no detectable fitness cost in most of the 1,344 growth conditions tested.

Isogenic plasmid-free and plasmid-containing clinical isolate from subjects 1 was tested. Raw data files generated by the Biolog PM reader (see Methods) are shown here and the data is summarized in Fig 5C. Bacterial cultures were inoculated into microarray plates and incubated for 48 hours, with cellular respiration (growth) recorded every 15 minutes. Each individual growth curve represents a unique growth condition. Yellow indicates no growth difference (plasmid-neutral), green indicates enhanced growth of plasmid-containing strains (plasmid advantage), and red indicates reduced growth in plasmid-containing strains (plasmid disadvantage). Phenotypic microarray plate number is indicated in the top left-hand cell. See[25] for a description of the stress or nutrient condition present in each well.

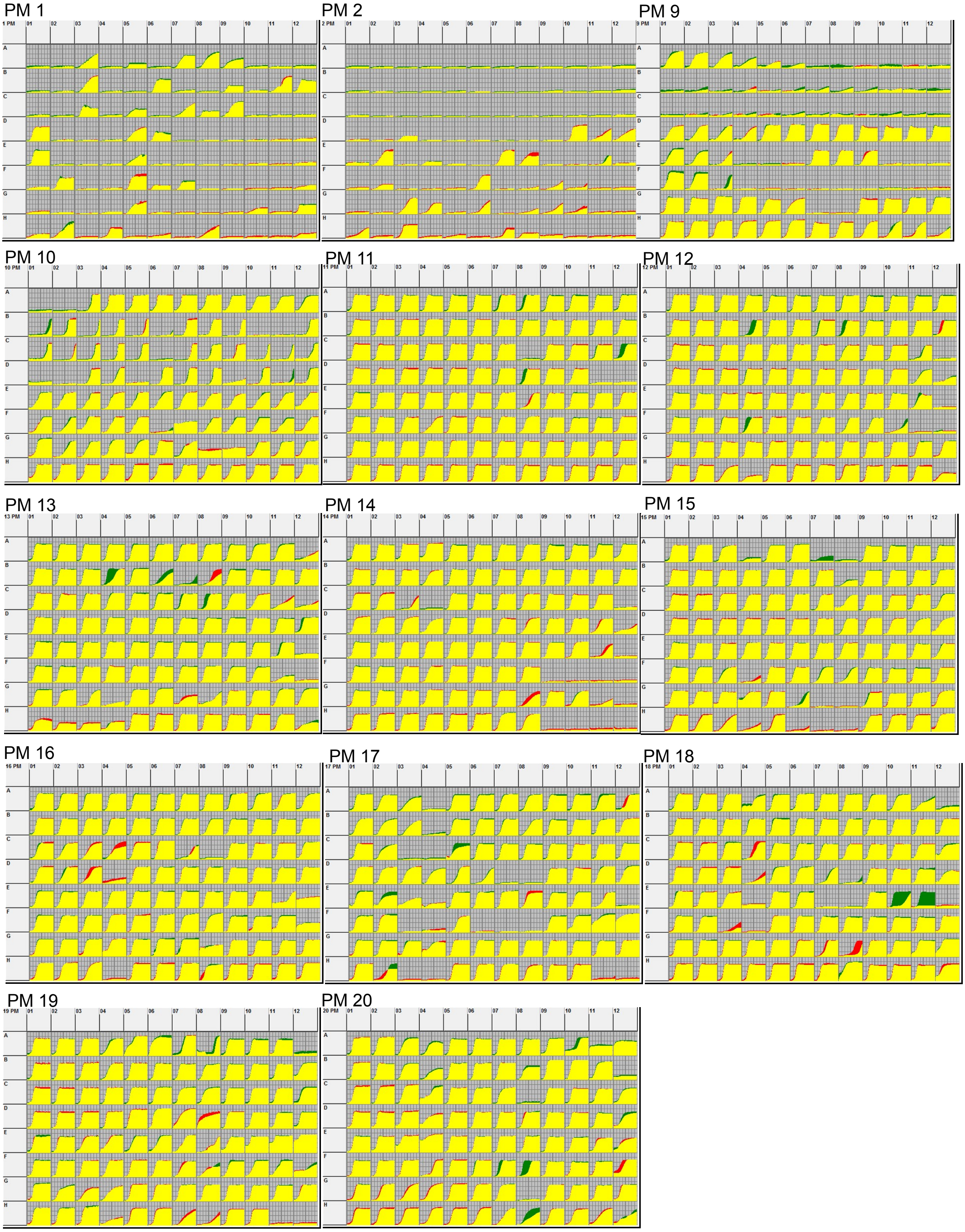

Supplementary Fig. 2. Phenotypic microarrays experiments show plasmid from subject 2 confer no detectable fitness cost in most of the 1,344 growth conditions tested.

Isogenic plasmid-free and plasmid-containing clinical isolate from subjects 2 was tested. Raw data files generated by the Biolog PM reader (see Methods) are shown here and the data is summarized in Fig 5C. Bacterial cultures were inoculated into microarray plates and incubated for 48 hours, with cellular respiration (growth) recorded every 15 minutes. Each individual growth curve represents a unique growth condition. Yellow indicates no growth difference (plasmid-neutral), green indicates enhanced growth of plasmid-containing strains (plasmid advantage), and red indicates reduced growth in plasmid-containing strains (plasmid disadvantage). Phenotypic microarray plate number is indicated in the top left-hand cell. See[25] for a description of the stress or nutrient condition present in each well.

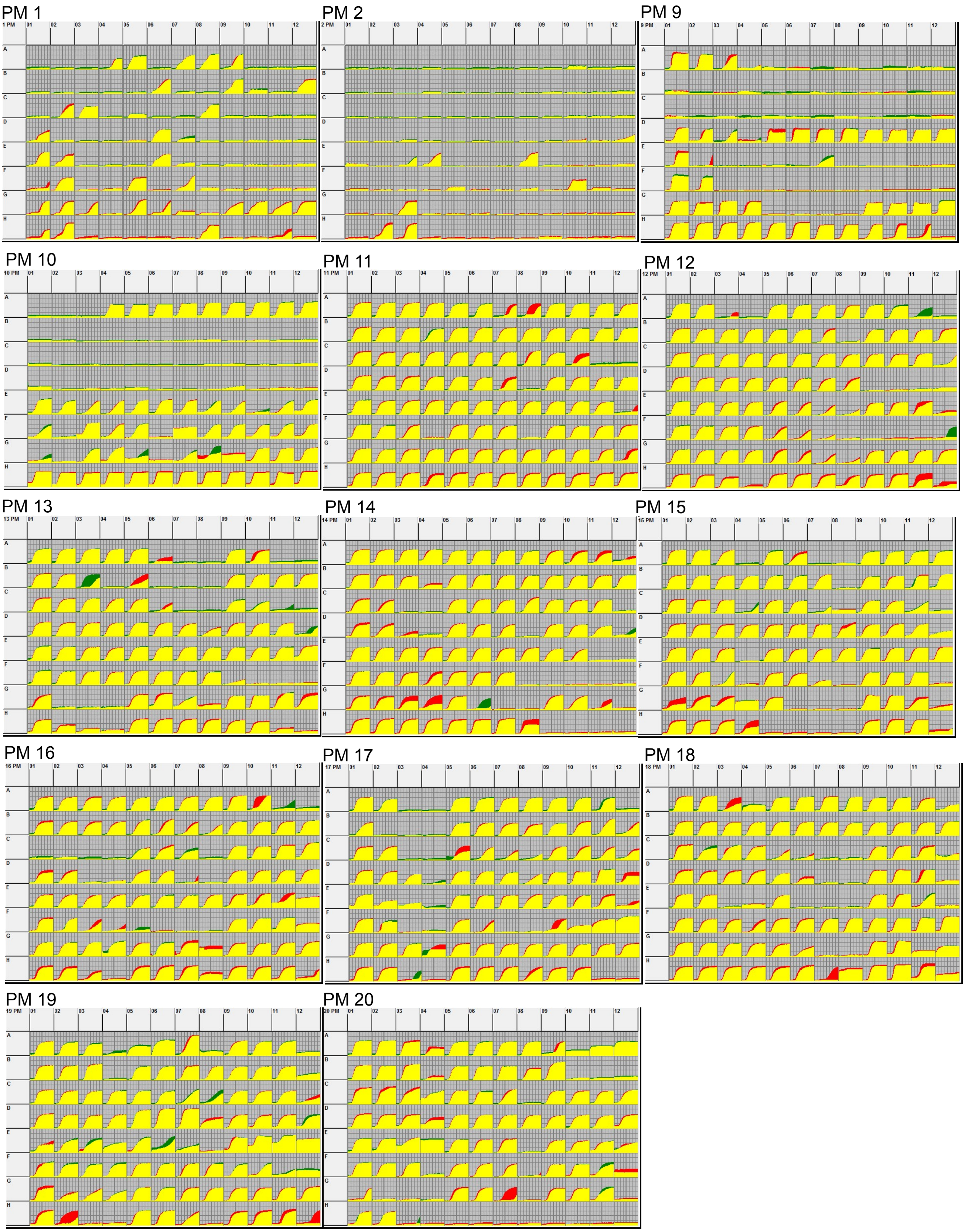

Supplementary Fig. 3. Phenotypic microarrays experiments show plasmid from subject 8 confer no detectable fitness cost in most of the 1,344 growth conditions tested.

Isogenic plasmid-free and plasmid-containing clinical isolate from subjects 8 was tested. Raw data files generated by the Biolog PM reader (see Methods) are shown here and the data is summarized in Fig 5C. Bacterial cultures were inoculated into microarray plates and incubated for 48 hours, with cellular respiration (growth) recorded every 15 minutes. Each individual growth curve represents a unique growth condition. Yellow indicates no growth difference (plasmid-neutral), green indicates enhanced growth of plasmid-containing strains (plasmid advantage), and red indicates reduced growth in plasmid-containing strains (plasmid disadvantage). Phenotypic microarray plate number is indicated in the top left-hand cell. See[25] for a description of the stress or nutrient condition present in each well.
