# Supplementary material for: Within-patient gene transfer between transiently and chronically infecting bacteria causes extreme antibiotic resistance during lung infections
Source: Nat Microbiol. 2026 Jul 23;11(8):2321–35. doi: 10.1038/s41564-026-02414-3 (PMC13423793; doi:10.1038/s41564-026-02414-3)
Supplement: Supplementary file 2 — Reporting Summary [file 41564_2026_2414_MOESM2_ESM.pdf]

nature portfolio

Corresponding author(s): Pradeep K. Singh

Last updated by author(s): May 3 2026

Reporting Summary

Nature Portfolio wishes to improve the reproducibility of the work that we publish. This form provides structure for consistency and transparency in reporting. For further information on Nature Portfolio policies, see our Editorial Policies and the Editorial Policy Checklist.

Statistics

For all statistical analyses, confirm that the following items are present in the figure legend, table legend, main text, or Methods section.

n/a	Confirmed	
☐	☒	The exact sample size (*n*) for each experimental group/condition, given as a discrete number and unit of measurement
☐	☒	A statement on whether measurements were taken from distinct samples or whether the same sample was measured repeatedly
☐	☒	The statistical test(s) used AND whether they are one- or two-sided<br>*Only common tests should be described solely by name; describe more complex techniques in the Methods section.*
☒	☐	A description of all covariates tested
☒	☐	A description of any assumptions or corrections, such as tests of normality and adjustment for multiple comparisons
☐	☒	A full description of the statistical parameters including central tendency (e.g. means) or other basic estimates (e.g. regression coefficient) AND variation (e.g. standard deviation) or associated estimates of uncertainty (e.g. confidence intervals)
☐	☒	For null hypothesis testing, the test statistic (e.g. *F*, *t*, *r*) with confidence intervals, effect sizes, degrees of freedom and *P* value noted<br>*Give P values as exact values whenever suitable.*
☒	☐	For Bayesian analysis, information on the choice of priors and Markov chain Monte Carlo settings
☒	☐	For hierarchical and complex designs, identification of the appropriate level for tests and full reporting of outcomes
☒	☐	Estimates of effect sizes (e.g. Cohen's *d*, Pearson's *r*), indicating how they were calculated

Our web collection on statistics for biologists contains articles on many of the points above.

Software and code

Policy information about availability of computer code

Data collection	llumina DNA sequencing data were collected using RTA v4.2.0. Reads were converted and demultiplexed using bcl2fastq v2.20. Oxford Nanopore sequencing data were collected using the MinKNOW, v23.04.5 - 24.11.10. Long reads were basecalled and processed using Guppy, v6.1.5 to 7.0.4. Bio-Rad's Quantasoft v3.1 was used to collect ddPCR data. BioTek Gen5 3.12 software was to collect OD600 measurements. Biolog File Management/Kinetic Analysis and Parametric Analysis software programs were used to collect data from Biolog Phenotypic Microarrays.
Data analysis	Statistical tests were performed using GraphPad Prism v10.5.0 (GraphPad Software, La Jolla, California)<br><br>Illumina reads were processed using the HTStream v1.3.3 pipeline (HTStream GitHub). Oxford Nanopore long reads were processed using FiltLong v0.2.1 (FiltLong GitHub). SPAdes v4.0.0 was used to generate whole genome contigs. Trycycler v0.5.5 was used to complete the genomes. Bakta v1.9.4 was used to annotate all assemblies. Kraken 2 was used to identify bacterial genera and species. Illumina reads were aligned to reference sequences using bwa-mem2 v2.2.155. LoFreq v2.1.5 was used for variant calling. MOB-suite v3.1.9, Bakta 1.9.4, oriTfinder2, and PlasAnn (https://plasann.rochester.edu/analysis) were used for plasmid annotation and typing. Toxin-antitoxin was determined using TADB v3.0. The phylogenetic trees were generated using IQ-TREE v2.3.6

For manuscripts utilizing custom algorithms or software that are central to the research but not yet described in published literature, software must be made available to editors and reviewers. We strongly encourage code deposition in a community repository (e.g. GitHub). See the Nature Portfolio guidelines for submitting code & software for further information.

Data

Policy information about availability of data

All manuscripts must include a data availability statement. This statement should provide the following information, where applicable:

- Accession codes, unique identifiers, or web links for publicly available datasets
- A description of any restrictions on data availability
- For clinical datasets or third party data, please ensure that the statement adheres to our policy

All sequencing data deposited at NCBI, BioProject number PRJNA1280179.
https://www.ncbi.nlm.nih.gov/bioproject/PRJNA1280179/

Research involving human participants, their data, or biological material

Policy information about studies with human participants or human data. See also policy information about sex, gender (identity/presentation), and sexual orientation and race, ethnicity and racism.

Reporting on sex and gender	N.A
Reporting on race, ethnicity, or other socially relevant groupings	N.A.
Population characteristics	N.A.
Recruitment	N.A.
Ethics oversight	Approval from the local Institutional Review Board was obtained by each site that collected bacterial isolates for this study.

Note that full information on the approval of the study protocol must also be provided in the manuscript.

Field-specific reporting

Please select the one below that is the best fit for your research. If you are not sure, read the appropriate sections before making your selection.

☒ Life sciences ☐ Behavioural & social sciences ☐ Ecological, evolutionary & environmental sciences

For a reference copy of the document with all sections, see nature.com/documents/nr-reporting-summary-flat.pdf

Life sciences study design

All studies must disclose on these points even when the disclosure is negative.

Sample size	Genomes of 277 bacterial samples were sequenced from 17 subjects. The sample size was determined by the availability of longitudinally stored isolates from subjects whose bacterial isolates exhibited extreme antibiotic resistance.
Data exclusions	No data were excluded in this study.
Replication	All experiments (MIC tests, plasmid copy number, conjugation frequency, plasmid stability, fitness, and hypermutator assay) were conducted with a minimum of three independent replicates, as detailed in the figure legends.
Randomization	No randomization was performed, as the aim of the study was to capture the emergence of resistant bacteria from the subjects' lungs.
Blinding	Blinding was not required in this study, as sequencing data from individual bacterial isolates collected longitudinally from each subject belonged to the same clone and consistently exhibited the same phenotype, either antibiotic-sensitive or resistant.

Reporting for specific materials, systems and methods

We require information from authors about some types of materials, experimental systems and methods used in many studies. Here, indicate whether each material, system or method listed is relevant to your study. If you are not sure if a list item applies to your research, read the appropriate section before selecting a response.

Materials & experimental systems

n/a	Involved in the study
☒	☐ Antibodies
☒	☐ Eukaryotic cell lines
☒	☐ Palaeontology and archaeology
☒	☐ Animals and other organisms
☒	☐ Clinical data
☒	☐ Dual use research of concern
☒	☐ Plants

Methods

n/a	Involved in the study
☒	☐ ChIP-seq
☒	☐ Flow cytometry
☒	☐ MRI-based neuroimaging

Plants

Seed stocks

N.A

Novel plant genotypes

N.A

Authentication

N.A
